# Etiology-specific prognostic value of ultra-early diffusion-weighted MRI after out-of-hospital cardiac arrest: a multicenter cohort study

**DOI:** 10.1186/s13054-026-05939-5

**Published:** 2026-03-06

**Authors:** Jin Hong Min, Yeonho You, Jung Soo Park, Changshin Kang, Hyun Shik Ryu, Wonjoon Jeong, Se Kwang Oh, So Young Jeon, In Ho Lee, Hye Seon Jeong, Sung Phil Chung, Rachel Beekman, Byung Kook Lee, Dong Hun Lee

**Affiliations:** 1 Department of Emergency Medicine, College of Medicine, Chungnam National University, 266, Munhwa-ro, Jung-gu, Daejeon, 35015 Republic of Korea; 2 Department of Emergency Medicine, Chungnam National University Hospital, 282, Munhwa-ro, Jung-gu, Daejeon, Republic of Korea; 3 Department of Emergency Medicine, Chungnam National University Sejong Hospital, 7, Bodam-ro, Sejong, Republic of Korea; 4 Department of Radiology, College of Medicine, Chungnam National University, 266, Munhwa-ro, Jung-gu, Daejeon, Republic of Korea; 5 Department of Neurology, Chungnam National University Hospital, 282, Munhwa- ro, Jung-gu, Daejeon, Republic of Korea; 6 Department of Emergency Medicine, Yonsei University College of Medicine, Seoul, Republic of Korea; 7 Department of Neurology, Yale School of Medicine, New Haven, CT USA; 8 Department of Emergency Medicine, Chonnam National University Medical School, Chonnam National University Hospital, 160, Baekseo-ro, Dong-gu, Gwangju, Republic of Korea

**Keywords:** Cardiac arrest, Diffusion-weighted MRI, Apparent diffusion coefficient, Hypoxic ischemic brain injury, Prognosis, Hypoxia

## Abstract

**Background:**

Diffusion-weighted magnetic resonance imaging (DW-MRI) within 0–6 h after return of spontaneous circulation can detect hypoxic-ischemic brain injury following out-of-hospital cardiac arrest (OHCA). Whether ultra-early findings differ by arrest etiology and how they should guide prognostication remains uncertain.

**Methods:**

We conducted a multicenter retrospective cohort study of OHCA survivors who underwent ultra-early DW-MRI (0–6 h); a subset had follow-up scans (72–96 h). Etiology was classified as cardiac or respiratory. We assessed the prognostic performance of qualitative ultra-early high-signal-intensity (HSI) and quantitative ADC-R(650) (% brain voxels with ADC ≤ 650 × 10⁻⁶ mm²/s) using receiver operating characteristic analysis to estimate the area under the curve (AUC) and sensitivity at 100% specificity. Qualitative HSI was based on routine clinical readings, with readers blinded to clinical outcomes and other clinical information. The primary outcome was poor neurological outcome at 6 months (CPC 3–5).

**Results:**

Among 176 patients (77 cardiac, 99 respiratory), 94 (53.4%) had poor outcomes. Ultra-early HSI occurred exclusively in patients with poor outcomes, yielding 100% specificity in both etiologies. At 100% specificity, sensitivity was significantly lower for respiratory etiology (52% vs. 86%; *P* = 0.006). Ultra-early HSI predicted poor outcome (AUC 0.80), with higher discrimination in the cardiac etiology subgroup (0.93 vs. 0.76; *P* < 0.001). In contrast, ultra-early ADC-R(650) showed modest prognostic value (AUC 0.77), but with similar discrimination between cardiac and respiratory etiology subgroups (0.80 vs. 0.77; *P* = 0.71). In the follow-up subset (*n* = 150), HSI demonstrated high discrimination for poor outcome (AUC 0.93) with no difference in AUC between cardiac and respiratory etiologies (0.96 vs. 0.95; *P* = 0.57). At this later time point, ADC-R(650) demonstrated high prognostic performance (AUC 0.91), with comparable results across etiologies (0.89 vs. 0.93; *P* = 0.47).

**Conclusions:**

HSI on ultra-early DW-MRI is specific for poor outcome after OHCA, but sensitivity is lower in respiratory etiology. DW-MRI at 72–96 h provides prognostic performance independent of etiology. Following OHCA, ultra-early HSI may help phenotype patients, particularly those with cardiac etiology, supporting an etiology-aware staged approach to DW-MRI–based prognostication. Further validation is warranted to explain delayed diffusion restriction in respiratory etiology.

**Graphical abstract:**

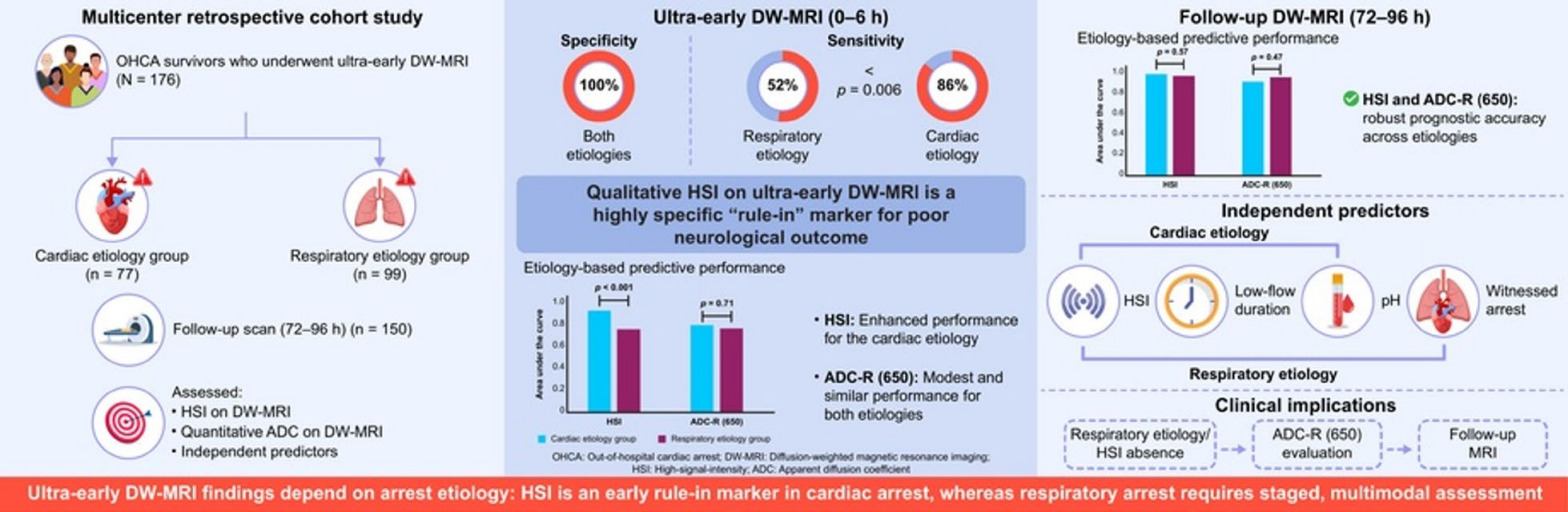

**Supplementary Information:**

The online version contains supplementary material available at 10.1186/s13054-026-05939-5.

## Background

Out-of-hospital cardiac arrest (OHCA) remains a major cause of mortality and long-term neurological disability [1, 2]. International guidelines recommend deferring formal neurological prognostication to ≥ 72 h after return of spontaneous circulation (ROSC) [3–5]. However, early-phase evaluation for hypoxic–ischemic brain injury (HIBI) is crucial for guiding family counseling, characterizing brain injury phenotypes, and avoiding premature prognostic conclusions that could contribute to withdrawal of life-sustaining treatment (WLST), particularly when critical care resources are limited [6].

Diffusion-weighted magnetic resonance imaging (DW-MRI) is among the most sensitive modalities for detecting HIBI [3–5]. Previous studies have emphasized obtaining DW-MRI during the subacute window (2–5 days after ROSC), when cytotoxic edema is most evident [7–11]. Ultra-early DW-MRI (≤ 6 h post-ROSC) can provide early prognostic information because visually detectable high-signal intensity (HSI) reflects the onset of cytotoxic edema. However, HSI is not consistently visible during the first hours after ROSC, which reduces the sensitivity of qualitative assessment alone [12–16].

Cardiac arrest is a heterogeneous syndrome; arrest characteristics, physiology, severity of brain injury, and neurologic outcome vary widely by etiology [17]. However, prior MRI studies have largely pooled cardiac and respiratory etiologies or restricted inclusion to presumed cardiac causes, implicitly assuming homogeneous brain injury trajectories and without formally comparing etiology-specific diffusion patterns or prognostic performance [12–16]. Whether ultra-early diffusion abnormalities differ by etiology, and how such differences should influence timing and interpretation of MRI-based prognostication, remains unclear.

Cardiac etiologies cause abrupt global brain ischemia with rapid development of cytotoxic edema, whereas respiratory etiologies result in a prolonged period of hypoxia and often hypercarbia preceding cardiac arrest. In experimental models, cerebral blood flow patterns differ between cardiac and respiratory arrest, with variations in the timing and regional distribution of hyperperfusion and hypoperfusion [18–23]. These pathophysiologic differences suggest that both the manifestation and prognostic significance of ultra-early DW-MRI findings may vary by arrest etiology, yet prior research has not clearly delineated this relationship.

Therefore, we aimed to compare the frequency and severity of ultra-early diffusion abnormalities between cardiac and respiratory etiologies and to evaluate the etiology-specific prognostic performance of qualitative HSI and quantitative apparent diffusion coefficient (ADC) thresholds at ultra-early and follow-up (72–96 h) time points. We also sought to characterize the temporal evolution of HIBI across etiologies using both qualitative and quantitative diffusion metrics. We hypothesized that ultra-early HSI would maintain high specificity for predicting poor outcome, but with variable sensitivity, depending on arrest etiology.

## Methods

### Study design and population

This multicenter, retrospective cohort study included data from patients with OHCA treated with targeted temperature management (TTM) at Chungnam National University Hospital (CNUH) and Chungnam National University Sejong Hospital (CNUSH) in Daejeon and Sejong, Korea, between May 2018 and September 2023. The study was approved by the institutional review boards of each participating hospital and conducted according to the guidelines of the Declaration of Helsinki (approval numbers: CNUH 2024-03-058 and CNUSH 2024-01-004). Clinical data were extracted without any personally identifiable information; therefore, the requirement for informed consent was waived.

**Fig. 1:**
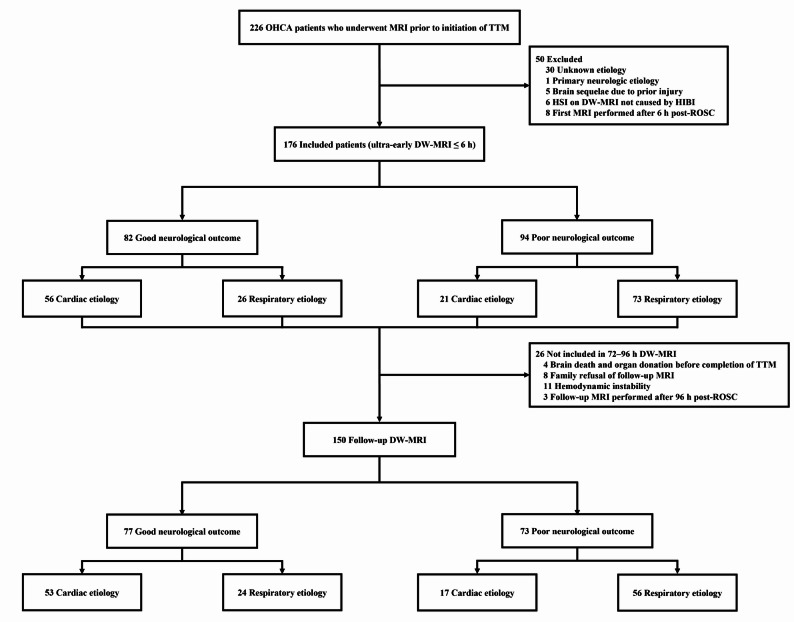
Flow diagram of patient enrollment. OHCA, out-of-hospital cardiac arrest; TTM, targeted temperature management; DW-MRI, diffusion-weighted MRI; ROSC, return of spontaneous circulation; ECMO, extracorporeal membrane oxygenation

Inclusion criteria comprised adult patients (≥ 18 years) with non-traumatic OHCA who underwent ultra-early DW-MRI at ≤ 6 h after ROSC. Exclusion criteria were as follows: (1) primary neurologic etiology such as stroke, subarachnoid hemorrhage, or encephalitis; (2) pre-existing brain injury; (3) HIS findings on DW-MRI attributable to causes other than HIBI (e.g., cerebral infarction); and (4) cases with indeterminate arrest etiology after diagnostic evaluation. A flow diagram summarizing patient selection and exclusion criteria is provided in Fig. 1.

The primary outcome was poor neurological status at 6 months after arrest, defined as a Cerebral Performance Category (CPC) score of 3–5. The CPC scale classifies patients into five categories: CPC 1 (good cerebral performance), CPC 2 (moderate disability), CPC 3 (severe disability), CPC 4 (vegetative state), and CPC 5 (brain death or death). Six-month CPC scores were prospectively assessed by an emergency physician or neurologist through in-person evaluations or structured telephone interviews.

### Data collection

We extracted the following data from each institution’s prospective registry: individual factors (age, sex, and Charlson Comorbidity Index [CCI]); cardiopulmonary resuscitation (CPR)-related clinical factors (witnessed arrest, bystander CPR, initial rhythm [shockable vs. non-shockable], etiology [cardiac vs. respiratory], and time from CPR to ROSC [low-flow time]); and post-arrest variables, including initial pH, initial serum lactate levels, revised post-cardiac arrest syndrome for therapeutic hypothermia score (rCAST) (Additional file 1: Table S1), fever occurrence, time-weighted average mean arterial pressure (TWA-MAP), and DW-MRI scan intervals (0–6 h and 72–96 h after ROSC).

Initial pH and serum lactate levels were obtained from the first arterial blood sample drawn immediately after ROSC. The ROSC-to-DW-MRI scan interval was defined as the time from ROSC to the start of imaging. Fever occurrence was defined as at least one body temperature measurement > 37.5 °C between ROSC and the ultra-early MRI scan [2]. Hypotension burden was summarized as the TWA-MAP from ROSC to the ultra-early MRI scan. TWA-MAP was calculated as the time-weighted mean of MAP from ROSC to ultra-early MRI by summing MAP × time between consecutive measurements and dividing by the total time [24, 25].

Arrest etiology was classified as cardiac or respiratory based on prehospital information, clinical assessment, diagnostic evaluation, and review of in-hospital records. Two investigators (J.H.M and J.S.P) independently reviewed all available data; in cases of disagreement, a third expert (Y.Y) adjudicated the final classification. Cases that could not be clearly assigned to either category were classified as indeterminate and excluded from the analyses. Cardiac etiology was defined as arrest resulting from a primary cardiac process—such as acute coronary syndrome or malignant arrhythmia—supported by findings from 12-lead electrocardiography, coronary angiography, and/or echocardiography, with no competing non-cardiac cause identified. Respiratory etiology was defined as arrest secondary to primary respiratory failure, including hypoxemic or hypercarbic respiratory failure, airway obstruction, aspiration, drowning, hanging, or pulmonary embolism, with clinical or laboratory evidence of respiratory compromise preceding arrest and no primary cardiac or neurological cause identified. These etiologic definitions and categorizations were based on current guideline definitions and previous reports comparing cardiac and respiratory causes of OHCA [3, 4, 17]. Inter-rater agreement statistics (e.g., Cohen’s κ) were not formally calculated; however, disagreements were infrequent and resolved by consensus with the third reviewer.

### Post–cardiac arrest care and neurological prognostication

Post–cardiac arrest care followed a standardized institutional protocol including TTM initiated early after ROSC, maintained at 33–36 °C for 24 h with controlled rewarming [26]. Sedation, shivering control, and seizure monitoring were standardized. Neurological prognostication was deferred until after rewarming and ≥ 72 h post-ROSC using a guideline-based multimodal approach. WLST was not performed during TTM unless brain death was confirmed, and MRI findings were not used in isolation for WLST decisions.

### MRI image processing and analysis

Ultra‑early DW-MRI was defined as brain MRI performed ≤ 6 h after ROSC. At each institution, the standard post–cardiac arrest care protocol recommends obtaining brain MRI at two time points—ultra‑early (0–6 h) and follow‑up (72–96 h) after ROSC—but imaging at either time point is not mandatory and is obtained as part of routine clinical care per institutional protocols. Accordingly, the official radiology report was uploaded to the electronic medical record and was available to the treating clinicians, who could also qualitatively review the images as part of usual care. MRI acquisition depends on clinical stability and is typically deferred during hemodynamic instability, ongoing procedures, or logistical limitations.

DW-MRI was acquired on 3.0-T scanners (Ingenia Elition X and Achieva; Philips Healthcare, Best, The Netherlands) using a single-shot spin-echo echo-planar imaging sequence (TR/TE, 4411.6/46.7 ms; slice thickness, 3 mm; interslice gap, 1 mm; field of view, 240 × 240 mm²; matrix, 128 × 126; number of signal averages, 1) with b-values of 0 and 1000 s/mm². T2-weighted imaging was also performed (TR/TE, 3000/80 ms; slice thickness, 5 mm; interslice gap, 1 mm; field of view, 220 × 220 mm²; matrix, 400 × 304; number of signal averages, 1). ADC maps were generated on the scanner console using a mono-exponential fit to the b = 0 and 1000 s/mm² images and exported in DICOM format.

HSI was defined as restricted diffusion, characterized by DW-MRI hyperintensity with concordant ADC hypointensity, involving the cerebral cortex and/or deep gray matter in a gyriform or diffuse pattern. To minimize overcalling physiological cortical hyperintensity, classification did not rely on relative cortical brightness alone; cortical signal changes were considered HSI only when a reproducible gyriform/diffuse pattern with corresponding ADC reduction clearly exceeded the expected physiological cortex–white matter contrast and could not be explained by physiological signal variation or partial-volume effects. Very limited or equivocal cortical hyperintensity confined to a single gyrus without a consistent gyriform pattern and ADC correlation was not classified as HSI. Deep gray matter–only diffusion restriction was classified as HSI (including unilateral cases) when it involved typical vulnerable structures (basal ganglia and/or thalami) with concordant ADC hypointensity and a non-territorial pattern consistent with hypoxic–ischemic injury; sharply demarcated territorial or embolic-appearing lesions were not classified as HSI and were considered more consistent with acute ischemic stroke [7, 12, 20]. Bilaterality was not required, and no strict numerical cutoff for the number of involved gyri was applied. A schematic summary of the qualitative pattern framework (including unilateral/bilateral cortical involvement, isolated deep gray matter involvement, and progression toward diffuse/global patterns) is provided in Supplementary Figure S3. Images were interpreted by board-certified neuroradiologists at CNUH (I.H.L.) for CNUH cases and by the Korea Tele-Radiology Reading Center (Seoul, Republic of Korea) for CNUSH cases; readers were aware that the scans were obtained after ROSC, but were blinded to clinical outcomes (including neurological outcomes) and had no access to other clinical information at the time of image interpretation.

Voxel-based ADC analysis was conducted using FSL (v5.0; FMRIB, Oxford, UK) following a previously validated protocol [27–30]. All DW-MRI datasets were exported from the clinical Picture Archiving and Communication System, converted into Neuroimaging Informatics Technology Initiative format, and imported into MRIcron software for analysis. To minimize scanner-related variability, ADC maps were harmonized using a standardized ComBat-based approach, followed by intensity distribution alignment. All preprocessing steps were performed in Python, and detailed harmonization procedures are provided in Additional file 2 (Figure S1). To reduce errors from artifacts, noise, and CSF partial-volume effects, voxels with ADC > 2000 × 10⁻⁶ mm²/s were excluded.

Threshold-based ADC quantification was performed by restricting analysis to brain parenchyma voxels with ADC values between 200 and 1200 × 10⁻⁶ mm²/s. Within this range, Mean ADC was defined as the voxel-wise global average ADC across the preprocessed brain parenchyma and summarized as median (interquartile range [IQR]) for analysis. For each threshold x, ADC-R(x) was defined as the percentage of brain parenchyma voxels with ADC ≤ x among voxels with ADC 200–1200 × 10⁻⁶ mm²/s. We used ADC-R(650) as the primary quantitative marker in prognostic models [27–29].

$$\begin{aligned}ADC-R\left(x\right)&=\frac{{Sum\,of\,voxels\,with\,ADC\,value}_{200}^{650}}{{Sum\,of\,voxels\,with\,ADC\,value}_{200}^{1200}}\\ & \quad\times100\end{aligned}$$

### Statistical analysis

Categorical variables were summarized as numbers (percentages), and continuous variables as medians with IQR given non-normal distributions. Categorical variables were compared using χ² tests (with continuity correction when appropriate) or Fisher’s exact tests, and continuous variables using the Mann–Whitney U test. For within-subject comparisons between ultra-early and follow-up DW-MRI in patients with paired measurements, the Wilcoxon signed-rank test was used.

Receiver-operating characteristic (ROC) curve analysis was used to evaluate the prognostic performance of imaging and clinical variables. The area under the ROC curve (AUC) with 95% confidence intervals (CIs) was calculated, and AUCs were compared using the DeLong test for ROC curves [31]. Paired or unpaired DeLong tests were applied as appropriate depending on whether ROC curves were derived from the same individuals. Sensitivity at 100% specificity was obtained by selecting, from the ROC curve, the cutoff(s) yielding specificity = 1.00 (i.e., no false positives observed in this cohort) and choosing the cutoff that maximized sensitivity under this constraint. For clinical interpretability, diagnostic indices (sensitivity, positive predictive value, and negative predictive value) were reported at this operating point (observed false-positive rate [FPR] = 0%).

Multivariable logistic regression models were constructed separately for cardiac and respiratory etiologies, with poor neurological outcome as the dependent variable. Ultra-early HSI and ADC-R(650) were included as primary imaging predictors, and all models were adjusted for prespecified clinical covariates (witnessed status, low-flow time, initial rhythm, initial pH, and initial lactate). Predictors were selected a priori based on prior literature and clinical plausibility, without data-driven variable selection [12, 28, 32]. When complete or quasi-separation occurred, ridge (L2-penalized) logistic regression was applied. Because ultra-early HSI was absent in all patients with good outcomes (complete separation), standard maximum-likelihood logistic regression can yield infinite estimates. Therefore, we used ridge-penalized logistic regression; coefficient estimates are shrunken and should be interpreted primarily as predictive weights rather than causal effect sizes. In glmnet, the penalty parameter (λ) was selected using 10-fold cross-validation to minimize binomial deviance within each etiology-specific model. Nonparametric bootstrap-based 95% CIs were generated using 500 resamples; calibration slope and intercept were obtained from bootstrap-based corrected estimates. There were no missing data for variables included in the multivariable models. All analyses were performed using IBM SPSS v27.0 (IBM Corp., Armonk, NY, USA), MedCalc v22.023 (MedCalc Software, Ostend, Belgium), Python (statsmodels, scikit-learn, numpy, pandas), and R (glmnet, boot, pROC).

## Results

### Baseline characteristics of the study cohort

Among the 226 OHCA patients who underwent brain MRI prior to initiation of TTM during the study period, 50 were excluded according to the predefined exclusion criteria (Fig. 1). A total of 176 patients were included in the final analysis (Fig. 1; Table 1). At 6 months, 82 (46.6%) and 94 (53.4%) patients had good and poor neurological outcomes, respectively. Between outcome groups, patients with poor outcomes were less likely to be male and less likely to have more favorable arrest characteristics (witnessed arrest, bystander CPR, shockable rhythm, and cardiac etiology), and they showed evidence of greater ischemic burden, including longer low-flow time, lower initial pH, higher lactate levels, and higher rCAST scores (all *P* < 0.05). No significant differences were observed in age, CCI, or ROSC-to-scan intervals at either the ultra-early or follow-up DW-MRI time point (Table 1). Initial pH values were generally biased toward acidosis rather than alkalosis in this cohort (overall median 7.14 [IQR 7.00–7.29]; Table 1).

**Table 1:** Baseline demographic data and cardiopulmonary resuscitation factors

Characteristic	Overall cohort (*N* = 176)	Good neurological outcome (*n* = 82)	Poor neurological outcome (*n* = 94)	*P*-value^a^	Cardiac etiology (*n* = 77)	Respiratory etiology (*n* = 99)	*P*-value^a^
Age, years	58.0 (43.5–68.8)	59.0 (39.8–66.5)	58.0 (44.5–70.0)	0.56	60.0 (48.0–67.0)	57.0 (39.0–69.0)	0.29
Male sex	131 (74.4)	67 (81.7)	64 (68.1)	0.04	67 (87.0)	64 (64.6)	< 0.001
CCI score	2.0 (1–4.0)	2.0 (0–4.0)	2.0 (1.0–4.0)	0.85	2.0 (1.0–4.0)	2.0 (0–4.0)	0.37
CPR-related clinical factors							
Witnessed arrest	114 (64.8)	71 (86.6)	43 (45.7)	< 0.001	61 (79.2)	53 (53.5)	< 0.001
Bystander CPR	133 (75.6)	71 (86.6)	62 (66.0)	0.002	61 (79.2)	72 (72.7)	0.32
Shockable rhythm	66 (37.5)	52 (63.4)	14 (14.9)	< 0.001	62 (80.5)	4 (4.0)	< 0.001
Cardiac etiology	77 (43.8)	56 (68.3)	21 (23.3)	< 0.001			
Low-flow time, min	20.0 (10.0–30.0)	13.0 (8.0–20.0)	27.0 (20.0–36.25)	< 0.001	18.0 (10.0–25.5)	23.0 (10.0–32.0)	0.12
Initial pH	7.14 (7.00–7.29)	7.23 (7.11–7.33)	7.08 (6.94–7.20)	< 0.001	7.21 (7.06–7.33)	7.10 (7.00–7.23)	0.003
Initial lactate, mmol/L	8.7 (5.8–11.0)	7.6 (4.2–10.9)	9.7 (6.9–11.5)	0.01	9.0 (5.2–11.0)	8.5 (6.1–11.0)	0.70
rCAST score	10.8 (7.0–15.0)	6.8 (3.9–10.5)	14.5 (12.0–16.0)	< 0.001	9.0 (5.0–12.0)	13.0 (8.5–16.0)	< 0.001
Time to target temperature, h	5.1 (4.2–6.8)	5.2 (4.2–6.9)	5.1 (4.0–6.7)	0.70	5.0 (4.2–6.7)	5.2 (4.0–6.8)	0.92
Fever occurrence, n (%)	9 (5.1)	7 (8.5)	2 (2.1)	0.08	4 (5.2)	5 (5.1)	1.00
TWA-MAP, mmHg	89.6 (83.7–97.5)	92.4 (82.9–99.5)	87.9 (83.8–96.6)	0.17	91.5 (84.6–97.7)	89.0 (82.2–97.5)	0.57
Ultra-early DW-MRI							
Scan-interval, h	2.6 (1.9–3.7)	2.4 (1.8–3.7)	2.7 (1.9–3.7)	0.56	2.5 (1.8–3.3)	2.7 (1.9–4.0)	0.56
HSI-presence	56 (31.8)	0	56 (59.6)	< 0.001	18 (23.4)	38 (38.4)	0.03
Mean ADC, ×10⁻⁶ mm²/s	776.8 (744.4–794.9)	789.1 (772.7–802.0)	755.3 (701.8–782.9)	< 0.001	782.6 (763.7–795.1)	769.3 (727.9–793.7)	0.02
ADC-R(650), %	17.5 (13.3–26.4)	14.9 (11.9–19.8)	23.8 (16.0–39.4)	< 0.001	16.7 (11.9–23.0)	18.3 (13.6–32.1)	< 0.001
Follow-up DW-MRI							
DW-MRI performed, n	150	77	73	–ᵇ	70	80	–ᵇ
Scan-interval, h	78.5 (76.6–81.3)	78.7 (76.9–82.0)	78.0 (76.4–80.5)	0.22	78.5 (76.5–81.2)	78.6 (76.6–81.5)	0.76
HSI-presence, n (%)	71 (47.3)	4 (5.2)	67 (91.8)	< 0.001	21 (30.0)	50 (62.5)	< 0.001
Mean ADC, ×10⁻⁶ mm²/s	802.3 (702.5–828.0)	824.6 (809.6–839.9)	703.6 (626.6–778.7)	< 0.001	819.0 (783.1–833.9)	750.8 (682.9–823.0)	< 0.001
ADC-R(650), %	16.7 (12.3–44.9)	13.0 (10.7–15.5)	45.4 (22.0–57.0)	< 0.001	14.4 (11.7–22.3)	32.9 (13.1–51.6)	< 0.001
CPC 5, n (%)	36 (20.5)	0	36 (38.3)	–ᵇ	4 (5.2)	32 (32.3)	–ᵇ
WLST after TTM	6	0	6	–ᵇ	2	4	–ᵇ

ᵃ P values are based on χ² tests for categorical variables and Mann–Whitney U tests for continuous variable

ᵇ P values were not calculated for CPC 5 and WLST after TTM because these variables are structurally dependent on CPC-based outcome grouping; for DW-MRI performed, P values were not calculated because follow-up imaging was not available for all patients

Data are presented as median (interquartile range) and n (%) for continuous and categorical variables, respectively

Fever occurrence was defined as at least one body temperature measurement > 37.5 °C between ROSC and the ultra-early MRI scan

Mean ADC was defined as the voxel-wise global average apparent diffusion coefficient across the preprocessed brain parenchyma and summarized as median (IQR) for analysis. ADC-R(650) was defined as the percentage of brain parenchyma voxels with ADC ≤ 650 × 10⁻⁶ mm²/s among voxels with ADC 200–1200 × 10⁻⁶ mm²/s

ADC, apparent diffusion coefficient; ADC-R(650), percentage of brain parenchyma voxels with ADC ≤ 650 × 10⁻⁶ mm²/s among voxels with ADC 200–1200 × 10⁻⁶ mm²/s; CCI, Charlson Comorbidity Index; CPR, cardiopulmonary resuscitation; DW-MRI, diffusion-weighted magnetic resonance imaging; HSI, high-signal intensity; IQR, interquartile range; rCAST, revised post-cardiac arrest syndrome score; TTM, targeted temperature management; TWA-MAP, time-weighted average mean arterial pressure

Etiologies were cardiac and respiratory in 77 (43.8%) and 99 (56.3%) patients, respectively. Arrests of respiratory etiology were characterized by fewer males, fewer witnessed arrests, a markedly lower rate of shockable rhythms, lower initial pH, and higher rCAST scores (all *P* ≤ 0.003). All other baseline characteristics, including bystander CPR, low-flow time, lactate, ROSC-to-scan intervals, age, and CCI, were comparable between the groups.

Fever occurrence and hypotension burden (TWA-MAP) between ROSC and ultra-early MRI were comparable between the good and poor neurologic outcome groups (Table 1) and were also similar between HSI-present and HSI-absent patients (Additional file 1: Table S2).

### Predictive performance of HSI on ultra-early DW-MRI

In the overall cohort, patients with poor outcomes more frequently showed HSI and had lower Mean ADC values and higher ADC-R(650) than those with good outcomes (Table 1). Although Mean ADC was slightly higher in cardiac etiology than in respiratory etiology overall, Mean ADC and ADC-R(650) did not differ significantly by etiology within either outcome group (Table 2). HSI was absent in all patients with good outcomes but present in 60% (56/94) of those with poor outcomes. Among poor outcome patients, HSI was more frequent in cardiac than in respiratory etiology (86% vs. 52%; *P* = 0.006; Table 2).

**Table 2:** Comparison of etiology amongst those with good versus poor outcomes

Characteristic	Good neurological outcome (*n* = 82)		*P*-value^a^	Poor neurological outcome (*n* = 94)		*P*-value^a^
	Cardiac etiology	Respiratory etiology		Cardiac etiology	Respiratory etiology	
Ultra-early DW-MRI						
DW-MRI performed, *n* = 176	56	26	–	21	73	–
HSI-presence	0	0	–	18 (86)	38 (52)	0.006
Mean ADC	786.1 (771.8–798.8)	793.3 (779.8–810.1)	0.12	762.7 (738.2–782.1)	746.8 (692.3–783.0)	0.45
ADC-R(650), %	15.0 (11.1–20.1)	14.5 (11.1–17.6)	0.49	24.1 (17.4–31.1)	23.6 (14.8–43.4)	0.89
	Good neurological outcome (*n* = 77)			Poor neurological outcome (*n* = 73)		
Follow-up DW-MRI						
DW-MRI performed, *n* = 150	53	24	–	17	56	–
HSI-presence	4 (8)	0	0.17	17 (100)	50 (89)	0.16
Mean ADC	821.7 (809.3–839.2)	826.9 (817.1–844.3)	0.20	735.7 (610.7–808.6)	697.1 (627.0–770.8)	0.34
ADC-R(650), %	13.6 (11.1–16.4)	12.0 (9.9–14.7)	0.13	39.0 (19.1–49.7)	46.0 (22.9–58.9)	0.38

ᵃ P values are based on χ² tests for categorical variables and Mann–Whitney U tests for continuous variables

Data are presented as median (interquartile range) and n (%) for continuous and categorical variables, respectively

Mean ADC was defined as the voxel-wise global average apparent diffusion coefficient across the preprocessed brain parenchyma and summarized as median (IQR) for analysis. ADC-R(650) was defined as the percentage of brain parenchyma voxels with ADC ≤ 650 × 10⁻⁶ mm²/s among voxels with ADC 200–1200 × 10⁻⁶ mm²/s

DW-MRI, diffusion-weighted magnetic resonance imaging; ADC, apparent diffusion coefficient; ADC-R(650), percentage of brain parenchyma voxels with ADC ≤ 650 × 10⁻⁶ mm²/s among voxels with ADC 200–1200 × 10⁻⁶ mm²/s; IQR, interquartile range; HSI, high-signal-intensity

**Fig. 2:**
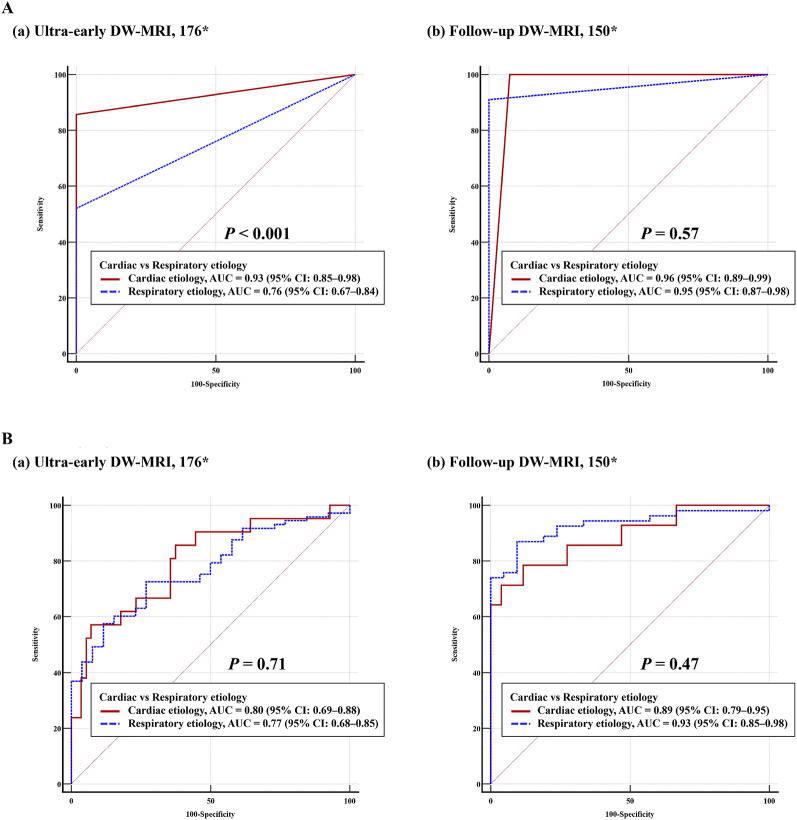
Receiver operating characteristic (ROC) curves of HSI presence and ADC-R(650) for predicting poor neurological outcome in ultra-early and follow-up DW-MRI. **A.** HSI on DW-MRI (a) Ultra-early DW-MRI subset: Discrimination was higher in cardiac etiology than in respiratory etiology (AUC 0.93 [95% CI 0.85–0.98] vs. 0.76 [95% CI 0.67–0.84]; *P* < 0.001). (b) Follow-up DW-MRI subset: Both etiologies demonstrated excellent and comparable predictive performance (AUC 0.96 [95% CI 0.89–0.99] vs. 0.95 [95% CI 0.87–0.98]; *P* = 0.57). **B.** ADC-R(650) on DW-MRI (a) Ultra-early DW-MRI subset: Predictive performance was comparable between cardiac and respiratory etiologies (AUC 0.80 [95% CI 0.69–0.88] vs. 0.77 [95% CI 0.68–0.85]; *P* = 0.71). (b) Follow-up DW-MRI subset: Diagnostic accuracy increased further, with slightly higher AUC in respiratory etiology than in cardiac etiology (AUC 0.93 [95% CI 0.85–0.98] vs. 0.89 [95% CI 0.79–0.95]; *P* = 0.47). AUCs were compared using the DeLong test. AUC, area under the ROC curve; CI, confidence interval; DW-MRI, diffusion-weighted magnetic resonance imaging; HSI, high-signal intensity; ADC-R(650), percentage of brain parenchyma voxels with ADC ≤ 650 × 10⁻⁶ mm²/s among voxels with ADC 200–1200 × 10⁻⁶ mm²/s (scaled per +10 percentage points)

Using HSI alone to predict poor outcome yielded an AUC of 0.80 (95% CI 0.73–0.86), with 60% sensitivity (49–70) at 100% specificity. Etiology-stratified analysis showed substantially higher predictive performance in cardiac etiology than respiratory etiology (AUC 0.93 [0.85–0.98] vs. 0.76 [0.67–0.84]), with corresponding sensitivities of 86% (64–97) and 52% (40–64) at 100% specificity (AUC difference *P* < 0.001) (Fig. 2A; Table 3).

**Table 3:** Diagnostic performance of HSI-presence in DW-MRI for predicting poor neurological outcome (overall and by etiology)

Group, *n*	AUC (95% CI)	Sensitivity (95% CI)	Specificity (95% CI)	PPV (95% CI)	NPV (95% CI)	TP	FP	TN	FN	*P*-value^b^
Ultra-early DW-MRI										
Overall, 176^a^	0.80 (0.73–0.86)	60 (49–70)	100 (96–100)	100 (94–100)	68 (63–73)	56	0	82	38	< 0.001
Cardiac etiology, 77 ^a^	0.93 (0.85–0.98)	86 (64–97)	100 (94–100)	100 (81–100)	95 (87–98)	18	0	56	3	< 0.001
Respiratory etiology, 99 ^a^	0.76 (0.67–0.84)	52 (40–64)	100 (87–100)	100 (91–100)	43 (30–56)	38	0	26	35	< 0.001
Follow-up DW-MRI										
Overall, 150 ^a^	0.93 (0.88–0.97)	92 (83–97)	95 (87–99)	94 (87–98)	92 (85–96)	67	4	73	6	< 0.001
Cardiac etiology, 70 ^a^	0.96 (0.89–0.99)	100 (81–100)	92 (82–98)	81 (62–92)	100	17	4	49	0	< 0.001
Respiratory etiology, 80 ^a^	0.95 (0.87–0.98)	89 (78–96)	100 (86–100)	100	80 (65–90)	50	0	24	6	< 0.001

^a^Number of patients included in the analysis

^b^P values indicate the statistical significance of the area under the ROC curve (AUC) compared with a null hypothesis of AUC = 0.5 (no discriminative ability)

AUC, area under the receiver operating characteristic curve; DW-MRI, diffusion-weighted magnetic resonance imaging; CI, confidence interval; PPV, positive predictive value; NPV, negative predictive value; TP, true positive; FP, false positive; TN, true negative; FN, false negative

For quantitative ADC analysis, ADC-R(650) demonstrated an AUC of 0.77 (95% CI 0.70–0.83), with 34% sensitivity (25–45) at 100% specificity. AUCs were comparable between cardiac and respiratory etiologies (0.80 [0.69–0.88] vs. 0.77 [0.68–0.85]; *P* = 0.71), with similar sensitivities at 100% specificity (24% vs. 37%) (Fig. 2B; Table 4).

**Table 4:** Diagnostic performance of ADC-R(650) for predicting poor neurological outcome (overall and by etiology)

Group, *n*	AUC (95% CI)	Cut-off value	Sensitivity (95% CI)	Specificity (95% CI)	PPV (95% CI)	NPV (95% CI)	TP	FP	TN	FN	*P*-value^b^
Ultra-early DW-MRI											
Overall, 176 ^a^	0.77 (0.70–0.83)	> 30.5%	34 (25–45)	100 (96–100)	100	57 (53–61)	32	0	82	62	< 0.001
Cardiac etiology, 77 ^a^	0.80 (0.69–0.88)	> 30.2%	24 (8–47)	100 (94–100)	100	78 (73–82)	5	0	56	16	< 0.001
Respiratory etiology, 99 ^a^	0.77 (0.68–0.85)	> 30.5%	37 (26–49)	100 (87–100)	100	36 (32–40)	27	0	26	46	< 0.001
Follow-up DW-MRI											
Overall, 150 ^a^	0.91 (0.85–0.95)	> 26.0%	72 (60–82)	100 (95–100)	100	79 (72–85)	52	0	77	21	< 0.001
Cardiac etiology, 70 ^a^	0.89 (0.79–0.95)	> 26.0%	64 (35–87)	100 (93–100)	100	91 (84–95)	11	0	53	6	< 0.001
Respiratory etiology, 80 ^a^	0.93 (0.85–0.98)	> 26.0%	74 (60–85)	100 (84–100)	100	60 (49–70)	41	0	24	15	< 0.001

^a^ Number of patients included in the analysis

^b^P values indicate the statistical significance of the area under the ROC curve (AUC) compared with a null hypothesis of AUC = 0.5 (no discriminative ability)

AUC, area under the receiver operating characteristic curve; CI, confidence interval; PPV, positive-predictive value; NPV, negative-predictive value; TP, true-positive; FP, false-positive; TN, true-negative; FN, false-negative; DW-MRI, diffusion-weighted magnetic resonance imaging; ADC, apparent diffusion coefficient; ADC-R(650), percentage of brain parenchyma voxels with ADC ≤ 650 × 10⁻⁶ mm²/s among voxels with ADC 200–1200 × 10⁻⁶ mm²/s

ADC-R(650) was defined as the percentage of brain parenchyma voxels with ADC ≤ 650 × 10⁻⁶ mm²/s among voxels with ADC 200–1200 × 10⁻⁶ mm²/s

### Follow-up (72–96 h) DW-MRI

Follow-up DW-MRI was available in 150 patients, including 77 (51%) and 73 (49%) with good and poor outcomes, respectively; 70 (47%) and 80 (53%) patients had cardiac and respiratory etiologies, respectively (Fig. 1). On follow-up DW-MRI, HSI was strongly associated with outcome, occurring in 92% of patients with poor outcomes (67/73) but only in 5% of those with good outcomes (4/77). Among patients with poor outcomes, HSI frequencies were similar between cardiac and respiratory etiologies (100% vs. 89%, *P* = 0.16). All false-positive HSI findings occurred in the cardiac subgroup (*n* = 4), whereas all false-negative cases occurred in the respiratory subgroup (*n* = 6) (Tables 2 and 3). On visual review, representative true-positive cases spanned a spectrum from subtle to overt diffusion abnormalities, as shown in Additional file 2 (Figures S2 and S3). In contrast, false-positive cases showed only limited, focal diffusion restriction without extensive cortical or deep gray matter involvement (Additional file 2: Figures S4 and Figure S5).

**Fig. 3:**
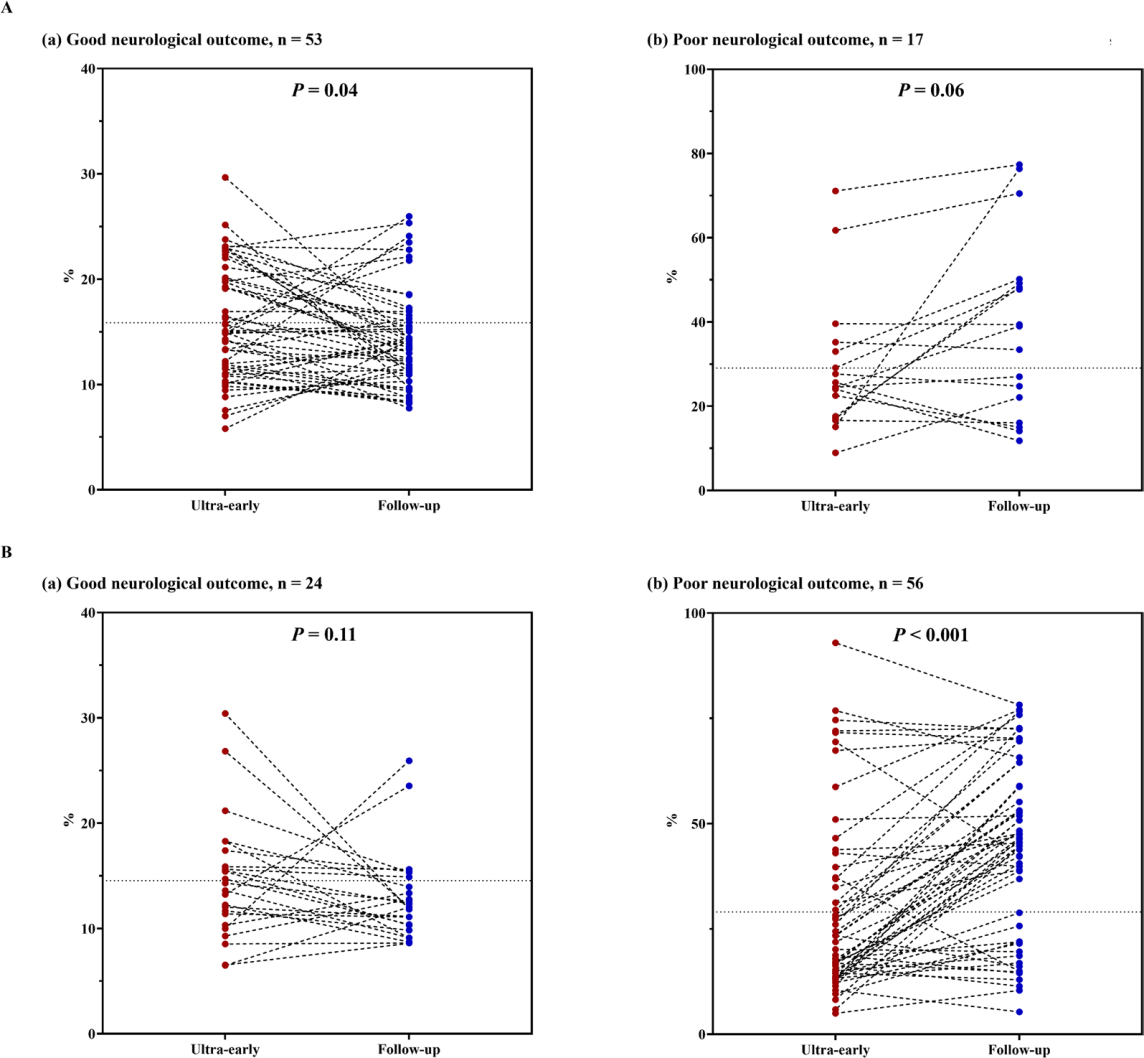
Longitudinal changes in ADC‑R(650) between ultra‑early and follow‑up DW‑MRI, stratified by etiology and neurological outcome. Paired dot-and-line plots depict within-patient trajectories of ADC-R(650) from ultra-early (0–6 h) to follow-up (72–96 h) DW-MRI within each etiology–outcome subgroup; values are presented as median (IQR) based on patients with paired scans. **A.** Cardiac etiology. (a) Good neurological outcome (*n* = 53): ADC-R(650) decreased from 15.0% (11.5–20.1) at ultra-early imaging to 13.6% (11.1–16.4) at follow-up (*P* = 0.04). (b) Poor neurological outcome (*n* = 17): ADC-R(650) increased from 24.5% (17.4–34.1) to 39.1% (19.1–49.7), but the change did not reach statistical significance (*P* = 0.06). **B**. Respiratory etiology. (a) Good neurological outcome (*n* = 24): ADC-R(650) showed a nonsignificant decrease from 14.0% (10.6–17.0) to 12.0% (9.9–14.7) (*P* = 0.11). (b) Poor neurological outcome (*n* = 56): ADC-R(650) increased from 21.1% (13.9–37.1) to 46.0% (22.9–58.9) (*P* < 0.001), consistent with progressive diffusion restriction in this subgroup. Ultra-early and follow-up measurements are shown as left and right points, respectively, connected by lines to indicate paired trajectories (ultra-early: red circles; follow-up: blue circles). P values were calculated using the Wilcoxon signed-rank test. DW-MRI, diffusion-weighted magnetic resonance imaging; ADC-R(650), percentage of brain parenchyma voxels with ADC ≤ 650 × 10⁻⁶ mm²/s among voxels with ADC 200–1200 × 10⁻⁶ mm²/s.

For quantitative ADC analysis, ADC-R(650) was significantly higher in patients with poor outcomes, both overall and within each etiology subgroup (all *P* < 0.001) (Additional file 2: Figure S6B). Longitudinally, ADC‑R(650) evolved differently according to etiology and outcome. In cardiac etiology, ADC‑R(650) decreased significantly over time in patients with good outcomes (*P* = 0.04) and increased without reaching statistical significance in those with poor outcomes (*P* = 0.06). In respiratory etiology, ADC‑R(650) showed a nonsignificant decrease in patients with good outcomes (*P* = 0.11) but increased significantly in those with poor outcomes (*P* < 0.001) (Fig. 3).

At follow-up, both HSI and ADC-R(650) showed high prognostic performance. HSI alone yielded AUCs of 0.96 (95% CI 0.89–0.99) and 0.95 (0.87–0.98) for cardiac and respiratory etiologies, respectively (*P* = 0.57) (Fig. 2). However, because four false-positive HSI findings occurred in the cardiac subgroup at this time point, etiology-specific sensitivities could not be compared using the same 0% FPR applied in the ultra-early analysis (Table 3). Similarly, the quantitative metric ADC-R(650) demonstrated strong, etiology-independent performance (AUC 0.89 [0.79–0.95] vs. 0.93 [0.85–0.98]; *P* = 0.47) (Table 4).

### Multivariable analysis stratified based on arrest etiology

**Fig. 4:**
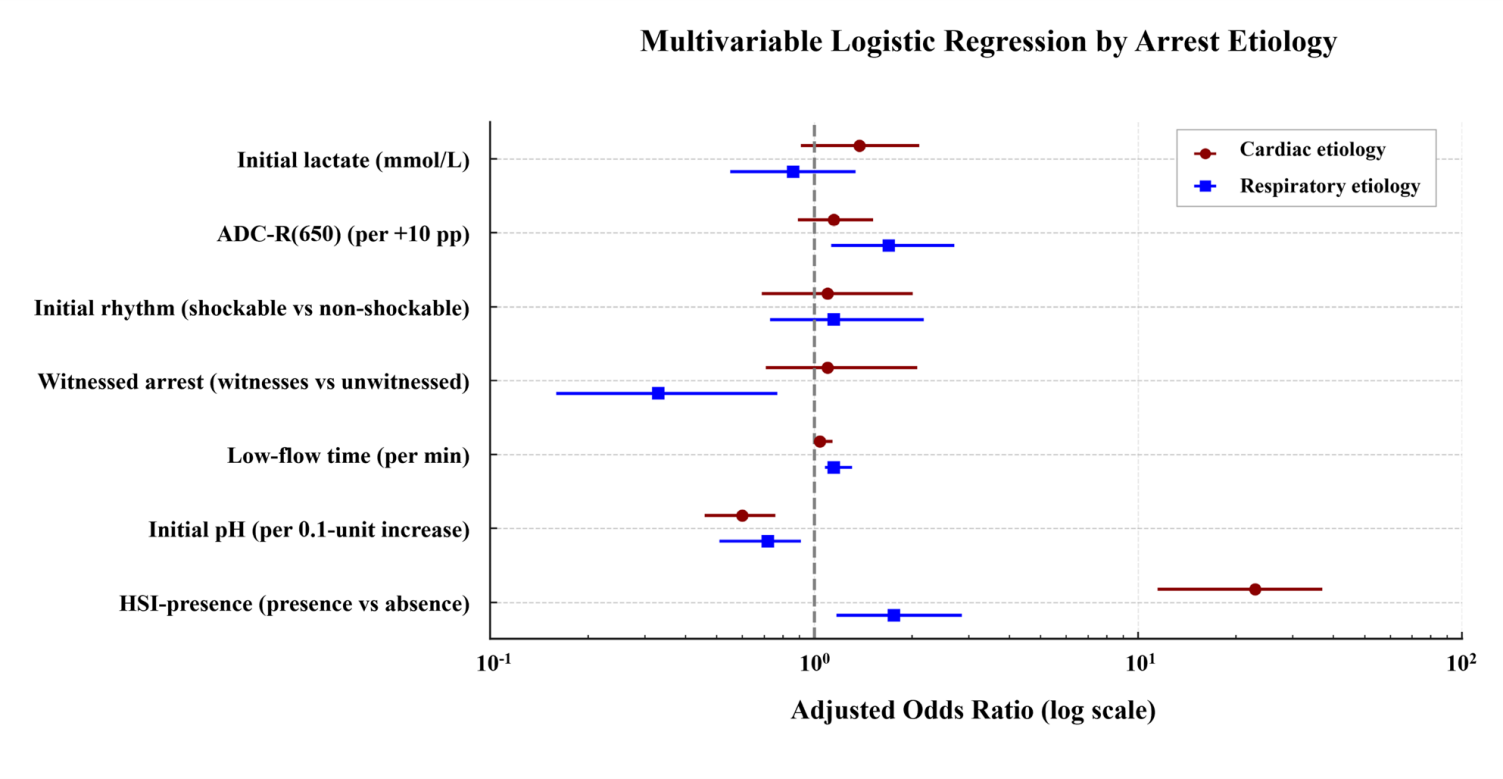
Multivariable logistic regression analysis for predictors of poor neurological outcome, stratified by arrest etiology Forest plots display adjusted odds ratios (aORs) with 95% confidence intervals for ultra-early predictors of poor neurological outcome within the cardiac and respiratory etiology subgroups. In cardiac etiology, the presence of HSI on ultra-early DW-MRI showed a strong association with poor outcome; longer low-flow time was associated with increased odds, whereas lower initial pH (greater acidosis) was associated with increased odds of poor outcome. In respiratory etiology, higher ADC-R(650) (scaled per + 10% points), presence of HSI, and longer low-flow time were associated with increased odds of poor outcome, whereas lower initial pH (greater acidosis) was associated with increased odds of poor outcome, and witnessed arrest were protective. Error bars indicate 95% confidence intervals. HSI, high-signal intensity on DW-MRI; DW-MRI, diffusion-weighted magnetic resonance imaging; ADC-R(650), percentage of brain parenchyma voxels with ADC ≤ 650 × 10⁻⁶ mm²/s among voxels with ADC 200–1200 × 10⁻⁶ mm²/s (scaled per + 10% points)

Using variables derived from ultra-early DW-MRI, we performed etiology-specific multivariable analyses to identify independent predictors of poor outcome (ridge-penalized logistic regression was used when separation occurred). In the cardiac etiology subgroup, ultra-early HSI was associated with increased odds of poor outcome (adjusted odds ratio [aOR] 23.0, 95% CI 11.5–37.0). Lower initial pH (greater acidosis) was associated with increased odds of poor outcome (aOR 0.60 per 0.1-unit increase, 95% CI 0.46–0.76), whereas longer low-flow time was associated with increased odds of poor outcome (aOR 1.04 per min, 95% CI 1.00–1.14). In the respiratory etiology subgroup, a greater diffusion burden measured by ADC-R(650) (aOR 1.70 per + 10% points, 95% CI 1.13–2.71), presence of HSI (aOR 1.76, 95% CI 1.17–2.86), and longer low-flow time (aOR 1.15 per min, 95% CI 1.08–1.31) were independently associated with increased odds of poor outcome, whereas lower initial pH (greater acidosis) was associated with increased odds of poor outcome (aOR 0.72 per 0.1-unit increase, 95% CI 0.51–0.91), and witnessed arrest (aOR 0.33, 95% CI 0.16–0.77) were protective (Fig. 4; Additional file 1: Table S3).

Model performance differed modestly across etiologies. The cardiac model demonstrated excellent discrimination (AUC 0.96 [0.89–0.99]) and low calibration error (Brier 0.050), with a sensitivity of 86% (69–100) at an FPR of 0%. The respiratory model also performed well (AUC 0.93 [0.86–0.97]; Brier 0.097), although sensitivity at a fixed specificity of 100% was lower (64%, 95% CI 54–92). Despite numerically higher discrimination and better calibration in the cardiac subgroup, the AUC difference was not statistically significant (DeLong, *P* = 0.34) (Additional file 1: Table S4; Additional file 2: Figure S7).

## Discussion

In this multicenter cohort of OHCA survivors treated with TTM, ultra-early DW-MRI revealed distinct etiology-specific differences in injury patterns and prognostic performance. Using qualitative HSI on ultra-early DW-MRI, respiratory etiology showed both lower discrimination and lower sensitivity than cardiac etiology (AUC 0.76 vs. 0.93; sensitivity at 100% specificity 52% vs. 86%), despite comparable low-flow times and ROSC-to-scan intervals. At follow-up, discrimination was similarly high across etiologies; however, etiology-specific sensitivities could not be compared at the same 0% FPR (100% specificity) because four false-positive HSI findings occurred exclusively in the cardiac subgroup. These false-positive cases were characterized by limited, low-burden diffusion restriction on follow-up imaging, suggesting that small focal HSI may occasionally be compatible with good outcome. Accordingly, ultra-early HSI may be considered an early, conservative rule-in prognostic marker (i.e., a highly specific finding for poor neurological outcome when present) in our cohort. Conversely, HSI absence on ultra-early DW-MRI—particularly in arrests of respiratory etiology—may not be reassuring and may warrant quantitative diffusion assessment and a planned follow-up MRI within a multimodal, staged prognostication strategy.

These etiology-specific differences may reflect fundamentally distinct injury mechanisms. Cardiac etiology arrest typically produces an abrupt no-flow state, resulting in rapid adenosine triphosphate depletion, synchronous cytotoxic edema, and early global diffusion restriction [22]. In contrast, respiratory etiology arrest evolves through progressively worsening hypoxemia ± hypercarbia and loss of autoregulation preceding circulatory collapse, generating mixed vasogenic and cytotoxic edema [18, 20–22, 33]. This combination may transiently elevate both T2 signal and ADC, producing pseudonormalization on ultra-early DW-MRI, consistent with the “T2 washout” phenomenon [23, 34]. These patterns align with preclinical comparisons of ventricular fibrillation and asphyxial arrest, in which residual perfusion delays the onset and regional extent of cytotoxic injury [18–21]. Clinically, early severe cerebral edema has been reported to be more common in non-cardiac etiologies, whereas presumed cardiac etiologies demonstrate more synchronous early cytotoxic injury with less vasogenic interference, facilitating clearer ultra-early diffusion restriction on DW-MRI (Additional file 2: Figure S8) [35].

These mechanistic differences were reflected in prognostic performance. In cardiac etiology, qualitative HSI on ultra-early DW-MRI showed excellent discrimination (AUC 0.93). In respiratory etiology, ultra-early HSI sensitivity was lower, and quantitative diffusion burden (ADC-R(650)) together with systemic severity markers (e.g., pH and low-flow time) provided complementary prognostic information. By 72–96 h, diffusion abnormalities converged between etiologies and prognostic accuracy was similarly high, underscoring complementary roles of ultra-early and follow-up MRI.

The etiology-stratified multivariable models further clarified how early imaging integrates with systemic markers. In cardiac etiology, ultra-early HSI remained a key predictor of poor outcome in the penalized models, and the overall model demonstrated excellent discrimination and good calibration. In respiratory etiology, no single marker was sufficient; higher ADC-R(650), the presence of HSI, and longer low-flow time were independently associated with increased odds of poor outcome, whereas lower initial pH was associated with increased odds of poor outcome, and witnessed arrest was protective. Despite lower sensitivity of HSI alone, the multivariable model achieved discrimination comparable to that of the cardiac etiology model. Taken together, these findings suggest that ultra-early diffusion restriction is more readily detectable in arrests of cardiac etiology, whereas in arrests of respiratory etiology early injury may be masked by mixed vasogenic and cytotoxic edema; therefore, absence of ultra-early HSI should not be interpreted as absence of brain injury.

These observations may help inform the potential clinical role of early imaging. In arrests of cardiac etiology, ultra-early DW-MRI demonstrated very high specificity for poor outcome in our cohort, as HSI within 0–6 h was associated with 0% observed FPR. However, these findings should be interpreted cautiously and require prospective multicenter external validation before broader clinical application. At the same time, the absence of ultra-early HSI may help identify patients with relatively preserved diffusion and potentially more salvageable brain injury, which could be useful for clinical-trial enrichment and phenotyping.

In arrests of respiratory etiology, a slower, regionally heterogeneous evolution of injury supports a staged approach that integrates ADC-R(650), systemic markers, and follow-up MRI. Neurological prognostication after cardiac arrest is recommended to be multimodal and delayed; within this context, our findings suggest that ultra-early DW-MRI provides information that is complementary to other early prognostic measures rather than definitive on its own. This complementary role of ultra-early MRI is supported by our prior single-center studies integrating early MRI with biomarkers and bedside examination. In our prior work using qualitative ultra-early DW-MRI, the presence of HSI within 0–6 h was associated with poor outcome with very high specificity (0% observed FPR), and combining DW-MRI with cerebrospinal fluid neuron-specific enolase (NSE) increased sensitivity while maintaining 100% specificity, supporting the concept of early imaging as a high-specificity “rule-in” component within a multimodal strategy [12]. In a separate ultra-early quantitative study, voxel-based ADC metrics improved prognostic modeling when integrated with early bedside examination (pupillary light reflex) and serum NSE, yielding improved discrimination (AUC 0.91) with preserved 0% FPR, further supporting that the incremental value of ultra-early MRI is best characterized in combination with complementary modalities [28].

This framework helps avoid premature prognostic conclusions and supports guideline-consistent, delayed multimodal prognostication after cardiac arrest [2–6]. Overall, our data suggest an etiology-aware, staged strategy in which ultra-early DW-MRI could provide early rule-in information, whereas follow-up DW-MRI and multimodal assessment may be warranted for arrests of respiratory etiology or when ultra-early HSI is absent [2, 5, 6, 27–29]. This proposed framework is hypothesis-generating and should be evaluated in future studies.

This study has several limitations. First, its retrospective design permits residual confounding despite harmonized imaging protocols and prespecified analyses. Second, despite clarification of our qualitative criteria, interpretation of subtle ultra-early HSI remains partly experience-dependent; thus, transferability to routine practice may be limited without further standardization and detailed guidance, particularly for less overt findings. Third, prognostication bias may have influenced treatment intensity or end-of-life decisions, as neuroimaging findings can affect clinicians’ expectations and communication with families. This risk was partly mitigated by institutional protocols that prohibited WLST during TTM. In addition, because poor neurologic outcome included death (CPC 5), any post-TTM WLST decisions could have contributed to outcome classification, and their impact cannot be fully disentangled from the prognostic value of ultra-early imaging findings. Nevertheless, some degree of self-fulfilling prophecy bias cannot be excluded [36]. Fourth, selection bias may have been introduced because patients with indeterminate arrest etiology and patients without ultra-early MRI were excluded. This approach reduced misclassification and strengthened the internal validity of etiology-specific comparisons but may have altered the apparent frequency of early HSI and limited the generalizability to populations with uncertain or mixed causes of arrest. Fifth, inter-rater reliability for etiology classification was not formally quantified. Nevertheless, etiology was determined through independent expert review and adjudication by a third reviewer, and only assigned when supported by unequivocal clinical and diagnostic evidence. Uncertain cases were intentionally classified as indeterminate to prioritize specificity over forced agreement. Sixth, MRI acquisition required hemodynamic stability and logistical availability, so our cohort likely under-represents the sickest patients with refractory shock or early death, which may have influenced the observed prevalence and prognostic performance of diffusion abnormalities. Seventh, although our prior single-center studies evaluated combinations of ultra-early MRI with biomarkers and bedside examination, the present multicenter analysis was not designed to systematically compare ultra-early MRI against the full set of guideline-recommended multimodal predictors (i.e., serial neurological examinations after exclusion of residual sedation and other confounders, electrophysiology including electroencephalography and somatosensory evoked potentials, and serial biomarker measurements such as NSE trends, alongside imaging). Therefore, we could not quantify the incremental value of ultra-early MRI beyond a complete guideline-based multimodal framework. Finally, the etiology-stratified multivariable models included several predictors relative to the number of outcome events, particularly in the cardiac subgroup; although ridge (L2-penalized) logistic regression and bootstrap internal validation were used to reduce overfitting, the resulting coefficients should be interpreted as descriptive and hypothesis-generating rather than definitive tools for individualized risk prediction.

This study has several strengths. To the best of our knowledge, it is the first study to examine etiology-specific ultra-early MRI signatures after cardiac arrest, integrating qualitative and quantitative diffusion markers to provide mechanistic and prognostic insights not attainable with a single parameter. Furthermore, reporting diagnostic indices at a 0% FPR highlights clinically conservative thresholds that are directly applicable to real-world neurological prognostication.

## Conclusions

This multicenter study demonstrates that diffusion abnormalities on brain MRI and their prognostic significance differ by arrest etiology and imaging time point. Ultra-early HSI strongly predicted poor outcome regardless of etiology; however, detection of diffusion restriction and its prognostic performance were reduced in patients with respiratory etiology. Follow-up MRI performed at 72–96 h provided high prognostic accuracy independent of etiology. Our findings support a potential role for ultra-early DW-MRI in phenotyping patients with cardiac arrest etiology, which may facilitate optimal patient selection for early interventions and improve identification of treatment responders. Prospective multicenter studies with time-resolved imaging protocols are needed to validate these findings and to elucidate the pathophysiologic mechanisms underlying delayed diffusion restriction in respiratory etiology.

## Supplementary Information

Supplementary Material 1. Tables S1–S4. Table S1. Calculation of rCAST. Table S2. Post-ROSC fever and hypotension prior to ultra-early DW-MRI, stratified by HSI status. Table S3. Multivariable logistic regression for predictors of poor neurological outcome. Table S4. Model performance metrics by arrest etiology. 

Supplementary Material 2. Figure S1–S8. Figure S1. Schematic representation of the harmonization process applied to ADC maps acquired from multiple MRI scanners. Figure S2. Representative spectrum of true-positive ultra-early DW-MRI findings, ranging from subtle to overt diffusion abnormalities. Figure S3. Schematic summary of qualitative HSI patterns on ultra-early DW-MRI. Figure S4. Four cases illustrating false-positive HSI findings on follow-up DW-MRI in patients with good neurological outcome. Figure S5. Evolution of diffusion abnormalities on ultra-early and follow-up DW-MRI according to neurological outcome. Figure S6. Quantitative ADC-R(650) comparison according to neurological outcome and etiology. Figure S7. Receiver operating characteristic (ROC) curves for the final multivariable prediction model for poor neurological outcome, stratified by arrest etiology. Figure S8. Conceptual schematic illustrating pathophysiological, perfusion, and DW-MRI differences between cardiac and respiratory arrest etiologies

## Data Availability

Anonymized data not published in this article can be made available upon reasonable request by any qualified investigator, subject to approval from the CNUH and CNUSH Institutional Review Board. The data supporting the findings of this study can be requested from the corresponding author, Jung Soo Park, at [cpcr@cnu.ac.kr](mailto: laphir@cnu.ac.kr).

## Abbreviations

ADC: Apparent diffusion coefficient
ADC-R(650): Percentage of brain parenchyma voxels with ADC ≤ 650 × 10⁻⁶ mm²/s among voxels with ADC 200–1200 × 10⁻⁶ mm²/s (scaled per + 10% points)
AUC: Area under the receiver operating characteristic curve
CCI: Charlson Comorbidity Index
CI: Confidence interval
CNUH: Chungnam National University Hospital
CNUSH: Chungnam National University Sejong Hospital
CPC: Cerebral Performance Category
CPR: Cardiopulmonary resuscitation
DW-MRI: Diffusion-weighted magnetic resonance imaging
FPR: False-positive rate
HIBI: Hypoxic–ischemic brain injury
HSI: High-signal intensity (restricted diffusion)
MRI: Magnetic resonance imaging
NSE: Neuron-specific enolase
OHCA: Out-of-hospital cardiac arrest
OR: Odds ratio
aOR: Adjusted odds ratio
PP: Percentage points
rCAST: Revised post-cardiac arrest syndrome score
ROC: Receiver operating characteristic
ROSC: Return of spontaneous circulation
TTM: Targeted temperature management
WLST: Withdrawal of life-sustaining treatment
