# Supplementary material for: Etiology-specific prognostic value of ultra-early diffusion-weighted MRI after out-of-hospital cardiac arrest: a multicenter cohort study
Source: Crit Care. 2026 Mar 6;30:145. doi: 10.1186/s13054-026-05939-5 (PMC13047829; doi:10.1186/s13054-026-05939-5)
Supplement: Supplementary file 1 — Supplementary Material 1. Tables S1–S4. Table S1. Calculation of rCAST. Table S2. Post-ROSC fever and hypotension prior to ultra-early DW-MRI, stratified by HSI status. Table S3. Multivariable logistic regression for predictors of poor neurological outcome. Table S4. Model performance metrics by arrest etiology. [file 13054_2026_5939_MOESM1_ESM.docx]

**Supplemental tables**

**Title: Etiology‑Specific Prognostic Value of Ultra‑Early Diffusion‑Weighted MRI After Out‑of‑Hospital Cardiac Arrest: A Multicenter Cohort Study**

**This file contains the following supplementary tables:**

**Table S1. Calculation of rCAST**

**Table S2. Post-ROSC fever and hypotension prior to ultra-early DW-MRI, stratified by HSI status**

**Table S3. Multivariable logistic regression for predictors of poor neurological outcome**

**Table S4. Model performance metrics by arrest etiology**

**Table S1.** Calculation of rCAST

| **Score** | **0** | **1** | **2** | **3** |
| --- | --- | --- | --- | --- |
| **Initial rhythm** | Shockable | Nonshockable |  |  |
| **Witness/until ROSC time** | <20 min | ≥20 min | No witness |  |
| **pH** | ≥7.31 | 7.30–7.16 | 7.15–7.01 | ≤7.00 |
| **Lactate** | ≤5.0 | 5.1–10.0 | 10.1–14.0 | ≥14.1 |
| **GCS M** | ≥2 | 1 |  |  |
| **Formula for calculation of the rCAST** | | | | |
| 1.0 × (initial rhythm score) + 2.0 × (witness/until ROSC time) + 2.5 × (pH score) + 0.5 × (lactate score) + 4.5 × (GCS M score) = rCAST points | | | | |

Abbreviations: rCAST, revised Postcardiac Arrest Syndrome for Therapeutic Hypothermia; ROSC, return of spontaneous circulation; GCS M, Motor Scale of the Glasgow Coma Scale

**Table S2.** Post-ROSC fever and hypotension prior to ultra-early DW-MRI, stratified by HSI status

|  | Overall cohort  (N=176) | HSI-absence  (n=120) | HSI-presence  (n=56) | *P*-value^a^ |
| --- | --- | --- | --- | --- |
| Fever occurrence, n (%) | 9 (5.1) | 8 (6.7) | 1 (1.8) | 0.28 |
| TWA-MAP, mmHg | 89.6 (83.7–97.5) | 94.5 (84.1–98.0) | 87.7 (82.1–96.3) | 0.15 |

Data are presented as median (interquartile range) and n (%) for continuous and categorical variables, respectively.

Fever occurrence was defined as at least one body temperature measurement >37.5 °C between ROSC and the ultra-early MRI scan.

Abbreviations: DW‑MRI, diffusion‑weighted magnetic resonance imaging; HSI, high‑signal intensity; TWA-MAP, time-weighted average mean arterial pressure

ᵃ P values are based on χ² tests or Fisher’s exact tests for categorical variables and Mann–Whitney U tests for continuous variables.

**Table S3**. Multivariable logistic regression for predictors of poor neurological outcome

| Variable | Adjusted OR (95% CI) | *P*-value | Clinical interpretation |
| --- | --- | --- | --- |
| Cardiac etiology (n = 77) |  |  |  |
| HSI-presence (yes vs. no) | 23.0 (11.5–37.0) | <0.001 | Associated with higher odds of poor outcome |
| ADC-R(650) (per +10 pp) | 1.15 (0.89–1.52) | 0.28 | Trend only |
| Witnessed arrest (yes vs. no) | 1.10 (0.71–2.08) | 0.48 | Not significant |
| Initial rhythm (shockable vs. non-shockable) | 1.10 (0.69–2.01) | 0.50 | Not significant |
| Low-flow time (per min) | 1.04 (1.00–1.14) | 0.02 | Longer low-flow worsens prognosis |
| Initial pH (per 0.1 unit increase) | 0.60 (0.46–0.76) | <0.001 | Lower pH associated with poor outcomes |
| Initial lactate (mmol/L) | 1.38 (0.91–2.11) | 0.09 | Not significant |
| Respiratory etiology (n = 99) |  |  |  |
| HSI-presence (yes vs. no) | 1.76 (1.17–2.86) | 0.02 | Independently associated with poor outcomes |
| ADC-R(650) (per +10 pp) | 1.70 (1.13–2.71) | 0.01 | A greater restricted volume predicts poor outcomes |
| Witnessed arrest (yes vs. no) | 0.33 (0.16–0.77) | 0.007 | Protective effect |
| Initial rhythm (shockable vs. non-shockable) | 1.15 (0.73–2.18) | 0.36 | Not significant |
| Low-flow time (per min) | 1.15 (1.08–1.31) | <0.001 | Strong risk factor |
| Initial pH (per 0.1 unit increase) | 0.72 (0.51–0.91) | 0.01 | Lower pH linked to poor outcomes |
| Initial lactate (mmol/L) | 0.86 (0.55–1.34) | 0.48 | Not significant |

Estimates are derived from ridge-penalized logistic regression due to separation; reported ORs are penalized (shrunken) and primarily reflect predictive weights.

Bootstrap CIs are provided; P-values should be interpreted with caution.

Abbreviations: ADC, apparent diffusion coefficient; CI, confidence interval; HSI, high-signal-intensity; OR, odds ratio

**Table S4**. Model performance metrics by arrest etiology

| Groups | AUC  (95% CI) | *P*-value | Brier score | Calibration slope (95% CI) | Calibration intercept (95% CI) | Sensitivity (95% CI) | Specificity (95% CI) | PPV (95% CI) | NPV (95% CI) | Nagelkerke R² | Hosmer–Lemeshow *P*-value | Akaike Information Criterion |
| --- | --- | --- | --- | --- | --- | --- | --- | --- | --- | --- | --- | --- |
| Cardiac model | 0.96 (0.89–0.99) | <0.001 | 0.050 | 1.02 (0.94–1.11) | –0.03 (–0.11 to 0.06) | 86 (69–100) | 100 | 100 (86–100) | 85 (70–100) | 0.81 | 0.48 | 92.6 |
| Respiratory model | 0.93 (0.86–0.97) | <0.001 | 0.097 | 0.93 (0.85–1.03) | 0.05 (–0.08 to 0.19) | 64 (54–92) | 100 | 100 (78–100) | 63 (44–86) | 0.68 | 0.22 | 118.4 |

Abbreviations: AUC, area under the receiver operating characteristic curve; CI, confidence interval; PPV, positive-predictive value; NPV, negative-predictive value

*Calibration slope and intercept were obtained from bootstrap-corrected logistic models (500 resamples).
