# Supplementary material for: Etiology-specific prognostic value of ultra-early diffusion-weighted MRI after out-of-hospital cardiac arrest: a multicenter cohort study
Source: Crit Care. 2026 Mar 6;30:145. doi: 10.1186/s13054-026-05939-5 (PMC13047829; doi:10.1186/s13054-026-05939-5)
Supplement: Supplementary file 2 — Supplementary Material 2. Figure S1–S8. Figure S1. Schematic representation of the harmonization process applied to ADC maps acquired from multiple MRI scanners. Figure S2. Representative spectrum of true-positive ultra-early DW-MRI findings, ranging from subtle to overt diffusion abnormalities. Figure S3. Schematic summary of qualitative HSI patterns on ultra-early DW-MRI. Figure S4. Four cases illustrating false-positive HSI findings on follow-up DW-MRI in patients with good neurological outcome. Figure S5. Evolution of diffusion abnormalities on ultra-early and follow-up DW-MRI according to neurological outcome. Figure S6. Quantitative ADC-R(650) comparison according to neurological outcome and etiology. Figure S7. Receiver operating characteristic (ROC) curves for the final multivariable prediction model for poor neurological outcome, stratified by arrest etiology. Figure S8. Conceptual schematic illustrating pathophysiological, perfusion, and DW-MRI differences between cardiac and respiratory arrest etiologies [file 13054_2026_5939_MOESM2_ESM.docx]

**Supplemental figures**

**Title: Etiology‑Specific Prognostic Value of Ultra‑Early Diffusion‑Weighted MRI After Out‑of‑Hospital Cardiac Arrest: A Multicenter Cohort Study**

**This file contains the following supplementary figures:**

**Figure S1. Schematic representation of the harmonization process applied to ADC maps acquired from multiple MRI scanners**

**Figure S2. Representative spectrum of true-positive ultra-early DW-MRI findings, ranging from subtle to overt diffusion abnormalities**

**Figure S3. Schematic summary of qualitative HSI patterns on ultra-early DW-MRI**

**Figure S4. Four cases illustrating false-positive HSI findings on follow-up DW-MRI in patients with good neurological outcome**

**Figure S5. Evolution of diffusion abnormalities on ultra-early and follow-up DW-MRI according to neurological outcome**

**Figure S6. Quantitative ADC-R(650) comparison according to neurological outcome and etiology**

**Figure S7. Receiver operating characteristic (ROC) curves for the final multivariable prediction model for poor neurological outcome, stratified by arrest etiology.**

**Figure S8. Conceptual schematic illustrating pathophysiological, perfusion, and DW-MRI differences between cardiac and respiratory arrest etiologies**

**Figure S1. Schematic representation of the harmonization process applied to ADC maps acquired from multiple MRI scanners**

Initially, spatial resampling (Step 1) was performed to ensure that all ADC maps shared a common spatial resolution and anatomical alignment, minimizing structural discrepancies across datasets. In Step 2, the voxel-wise ADC values from each resampled image were flattened into one-dimensional arrays to facilitate statistical batch effect correction. Step 3 involved ComBat harmonization, a method used to adjust for scanner-dependent variability while preserving biological signals, reducing artificial intensity differences introduced by hardware and acquisition protocols. Step 4 involved histogram matching, where the voxel intensity distribution of each harmonized ADC map was aligned to that of a predefined reference map, standardizing the intensity scaling across patients. The resulting harmonized ADC dataset enabled robust cross-scanner comparability, reducing confounding technical variation and supporting accurate group-level analyses. ADC, apparent diffusion coefficient.

Abbreviations: ADC, apparent diffusion coefficient; MRI, magnetic resonance imaging

**
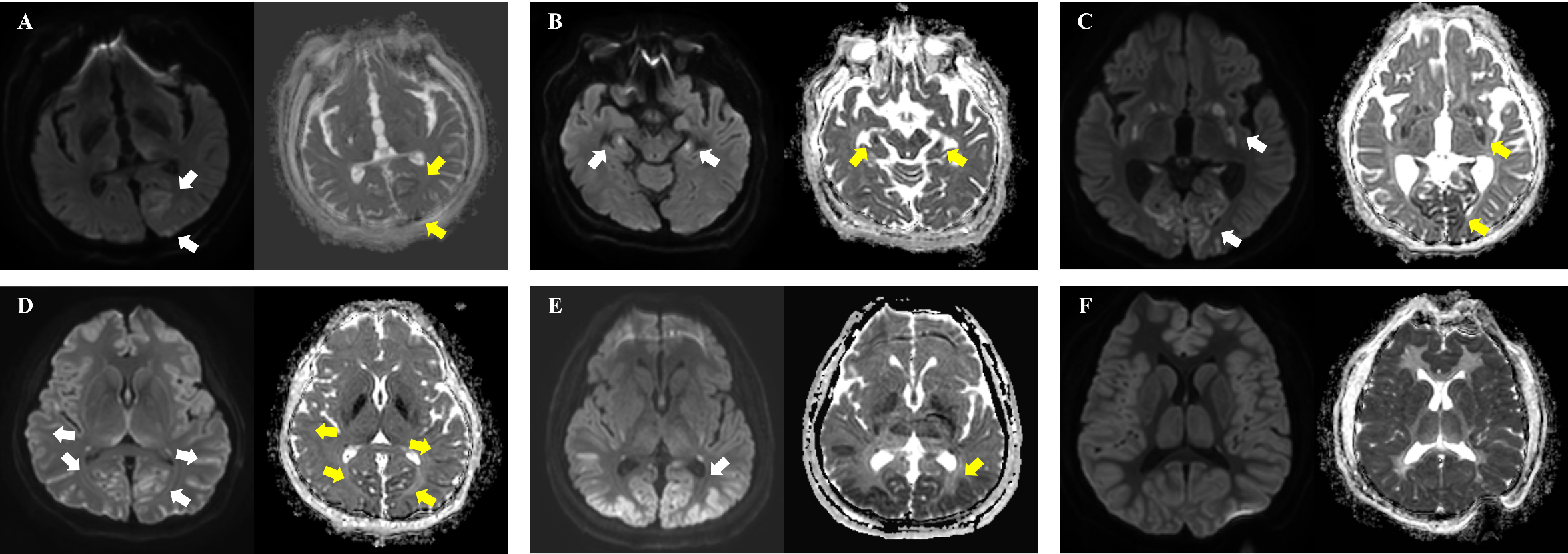
**

**Figure S2. Representative spectrum of true-positive ultra-early DW-MRI findings, ranging from subtle to overt diffusion abnormalities**

Panels A–C illustrate relatively subtle or limited HSI, whereas panels D–F show progressively more conspicuous and extensive HSI. Although a single representative axial slice is shown for each panel, HSI was confirmed on consecutive adjacent slices (multislice continuity). White arrows indicate HSI on DWI, and yellow arrows indicate corresponding ADC hypointensity.

**A.** 51-year-old female Limited unilateral occipital gyriform cortical HSI with concordant ADC hypointensity.

**B.** 59-year-old male: Limited bilateral hippocampal HSI with concordant ADC hypointensity.

**C.** 74-year-old male: Multi-regional HSI involving the bilateral basal ganglia and the occipital cortex, with concordant ADC hypointensity.

**D.** 51-year-old male: Predominant unilateral hippocampal HSI with concordant ADC hypointensity.

**E.** 33-year-old male: Diffuse cortical HSI involving the parietal and occipital cortices, with concordant ADC hypointensity.

**F.** 36-year-old male: Diffuse and extensive HSI involving large portions of the cerebral cortex with prominent deep gray matter involvement, with concordant ADC hypointensity.

Abbreviations: DW-MRI, diffusion-weighted magnetic resonance imaging; ADC, apparent diffusion coefficient; HSI, high signal intensity


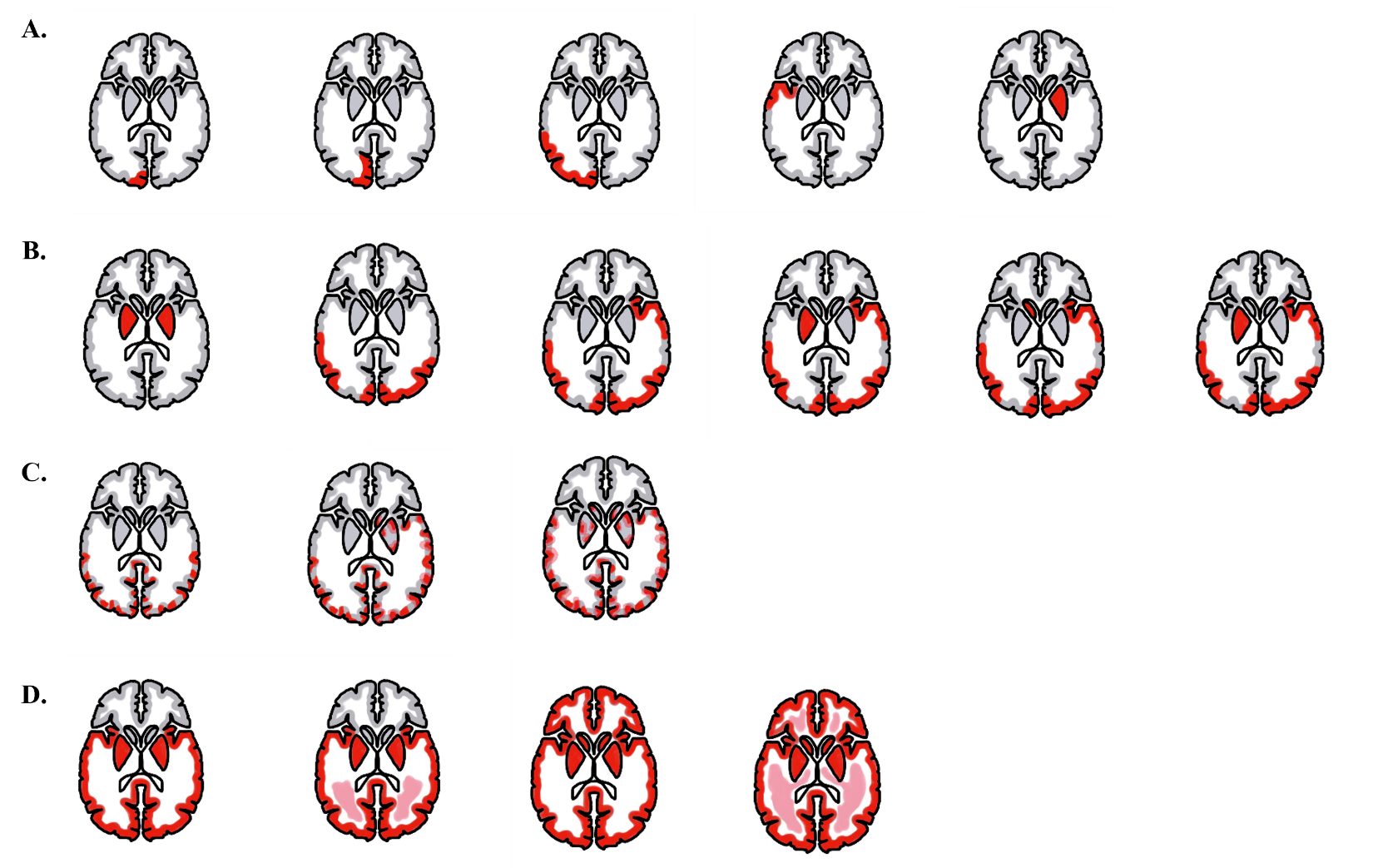


**Figure S3. Schematic summary of qualitative HSI patterns on ultra-early DW-MRI**

This schematic illustrates the qualitative framework used to categorize HSI on ultra-early diffusion-weighted MRI according to distribution and extent of diffusion restriction. HSI was defined as DWI hyperintensity with concordant ADC hypointensity and was confirmed on consecutive adjacent slices (multislice continuity) to minimize misclassification of artifactual or physiological cortical hyperintensity. Red shading indicates regions considered consistent with restricted diffusion only when accompanied by concordant ADC hypointensity (i.e., true diffusion restriction rather than physiological cortical hyperintensity).

**A.** Unilateral gyriform cortical involvement and isolated deep gray matter involvement (basal ganglia/thalami; unilateral) with minimal extent.

**B.** Isolated deep gray matter involvement (basal ganglia/thalami; bilateral) and early bilateral cortical involvement with limited-to-moderate extent.

**C.** Wider bilateral multiple cortical involvement with combined cortical and deep gray matter involvement.

**D.** Diffuse and extensive cortical involvement, often with prominent deep gray matter involvement, representing the most extensive pattern on the spectrum.

Suong Won Ahn created the illustrations.

Abbreviations: DW-MRI, diffusion-weighted magnetic resonance imaging; HSI, high signal intensity


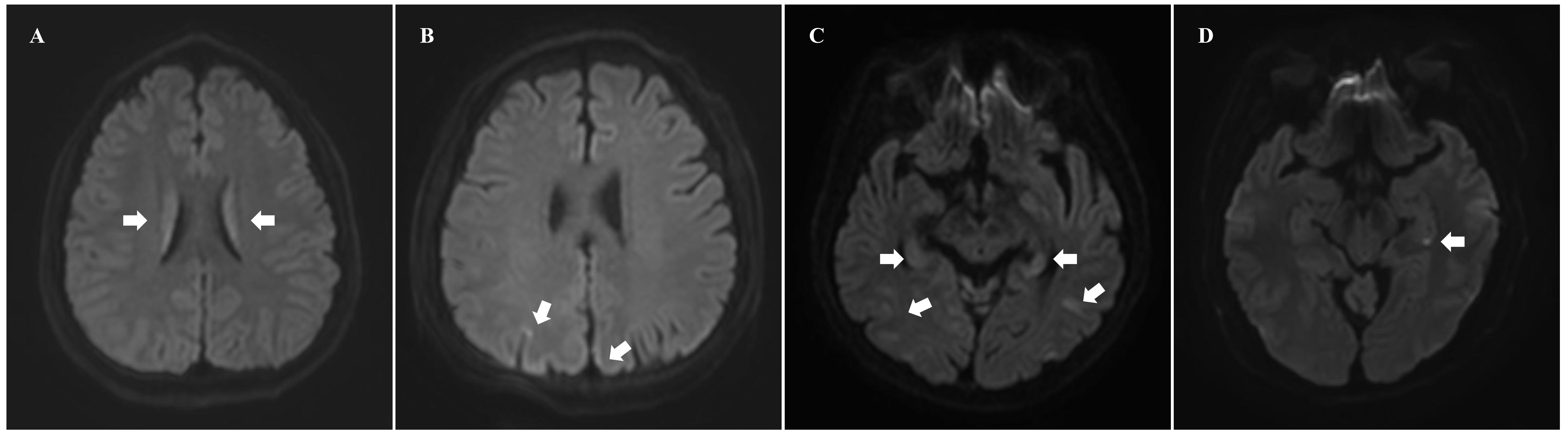


**Figure S4. Four cases illustrating false-positive HSI findings on follow-up DW-MRI in patients with good neurological outcome**

All cases demonstrated limited, focal diffusion restriction without any extensive cortical or deep gray matter involvement.

**A.** 46-year-old male: HSI in both periventricular regions.

**B.** 57-year-old male: HSI in both parietal lobes.

**C.** 58-year-old male: HSI in both hippocampi with suspected involvement of both temporo-occipital lobes.

**D.** 45-year-old male: HSI in the left hippocampus.

Abbreviations: DW-MRI, diffusion-weighted magnetic resonance imaging; ADC, apparent diffusion coefficient; HSI, high signal intensity

**
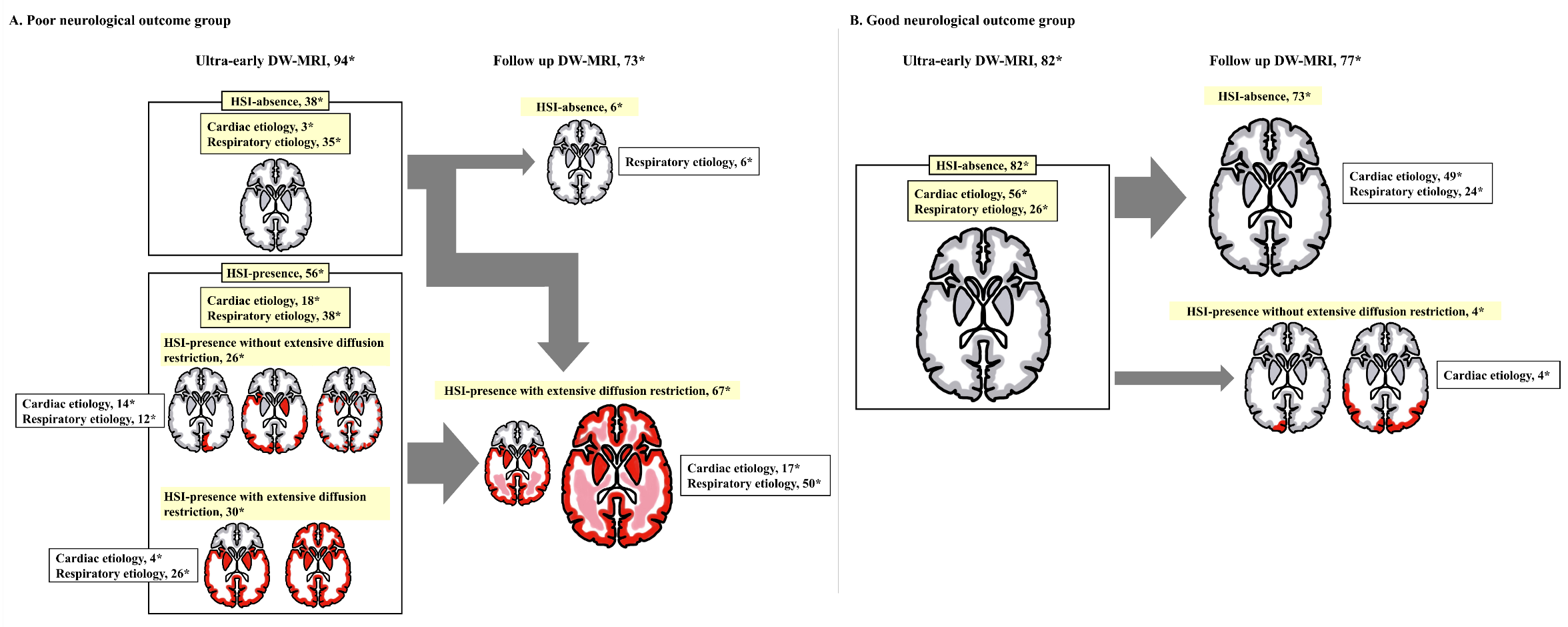
**

**Figure S5. Evolution of diffusion abnormalities on ultra-early and follow-up DW-MRI according to neurological outcome**

**A. Poor neurological outcome group**

Flow diagram demonstrating findings on ultra-early DW-MRI (n=94) and 72–96 h DW-MRI (n=73)

Patients were categorized by the presence of high signal intensity (HSI) and by the presence of extensive diffusion restriction. Among those with HSI on ultra-early imaging (n=56), many progressed to extensive diffusion restriction on follow-up MRI. Some patients without initial HSI developed new HSI on follow-up imaging. Cardiac and respiratory etiologies are reported at each step. Differences in the number of patients between imaging time points reflect that not all patients underwent follow-up DW-MRI. **B. Good neurological outcome group**

Flow diagram showing changes in HSI between ultra-early DW-MRI (n=82) and follow-up DW-MRI (n=77)

Most patients had no HSI at either time point. A small number of patients developed limited HSI without extensive diffusion restriction on follow-up DW-MRI. Etiologic distributions are shown for each transition.

* indicates the number of patients (n) for each category and subgroup.

Abbreviations: DW-MRI, diffusion-weighted magnetic resonance imaging; HSI, high signal intensity


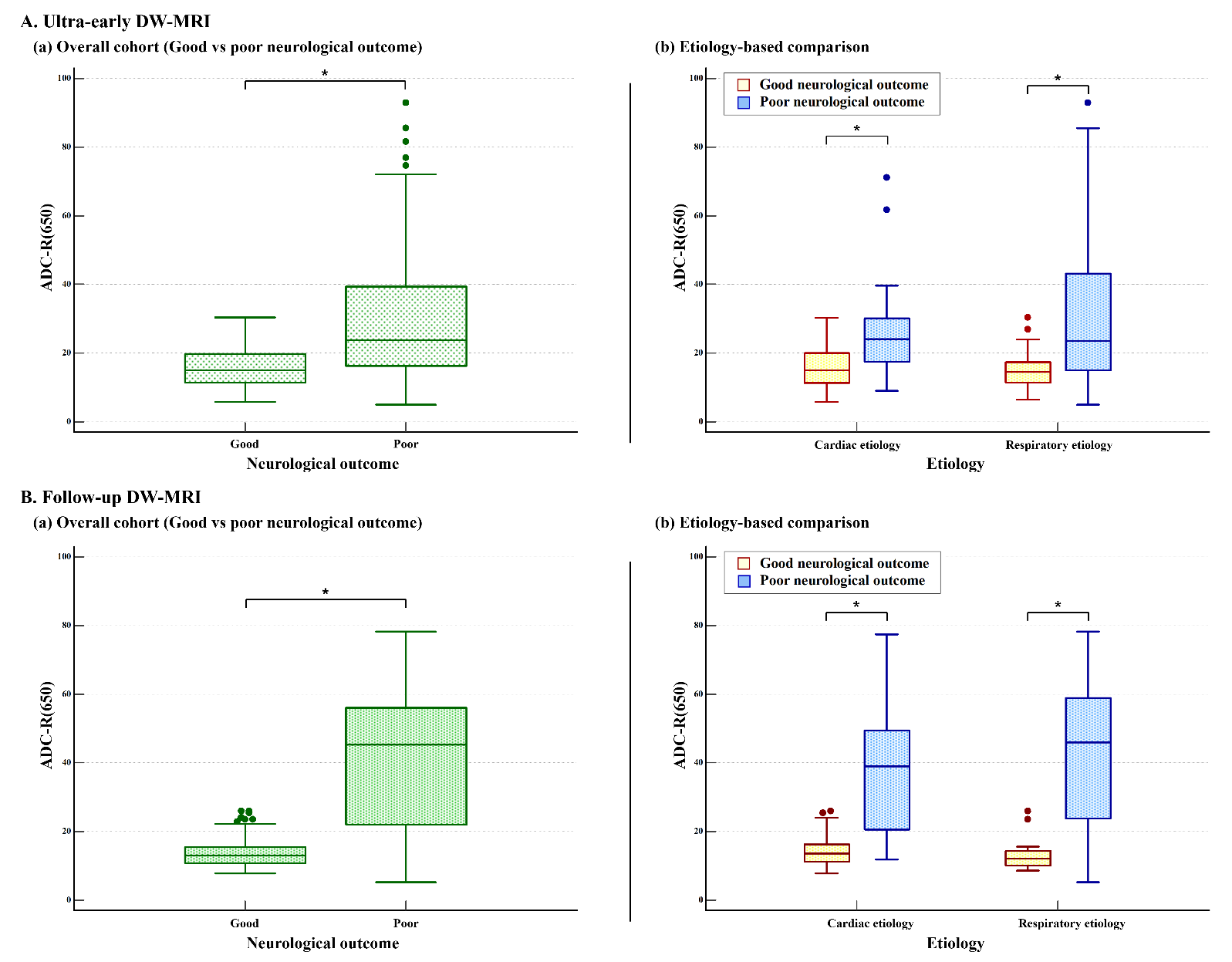


**Figure S6. Quantitative ADC-R(650) comparison according to neurological outcome and etiology**

**A. Ultra-early DW-MRI** (a) Box-and-whisker plots showing ADC-R(650) values in the overall cohort, stratified based on neurological outcome

Patients with poor outcomes exhibited significantly higher ADC-R(650) percentages than those with good outcomes *(P <* 0.001). (b) Etiology-based comparison of ADC-R(650) values for cardiac and respiratory arrest

Within each etiology, patients with poor outcomes showed significantly elevated ADC-R(650) compared with those with good outcomes (*P* < 0.001). The distribution of ADC-R(650) was wider in respiratory arrest, reflecting greater heterogeneity in diffusion changes. **B. Follow-up DW-MRI** (a) In the overall cohort, ADC-R(650) values were markedly higher in patients with poor neurological outcome than in those with good outcome (*P* < 0.001), with greater separation than in the ultra-early phase. (b) Etiology-based comparison at follow-up

Both cardiac and respiratory arrest groups demonstrated significantly higher ADC-R(650) values among patients with poor outcomes (*P* < 0.001), with consistently larger effect sizes than in the ultra-early phase.

Boxplots indicate median, IQR, and full data range; outliers are shown as individual points. ADC-R(650) is expressed as the percentage of brain voxels with ADC ≤ 650× 10⁻⁶ mm²/s. ***** denotes statistically significant differences (*P* < 0.001).

Abbreviations: DW-MRI, diffusion-weighted magnetic resonance imaging; ADC, apparent diffusion coefficient; ADC-R(650), percentage of brain parenchyma voxels with ADC ≤ 650 × 10⁻⁶ mm²/s (200–1200 × 10⁻⁶ mm²/s range); IQR, interquartile range

**
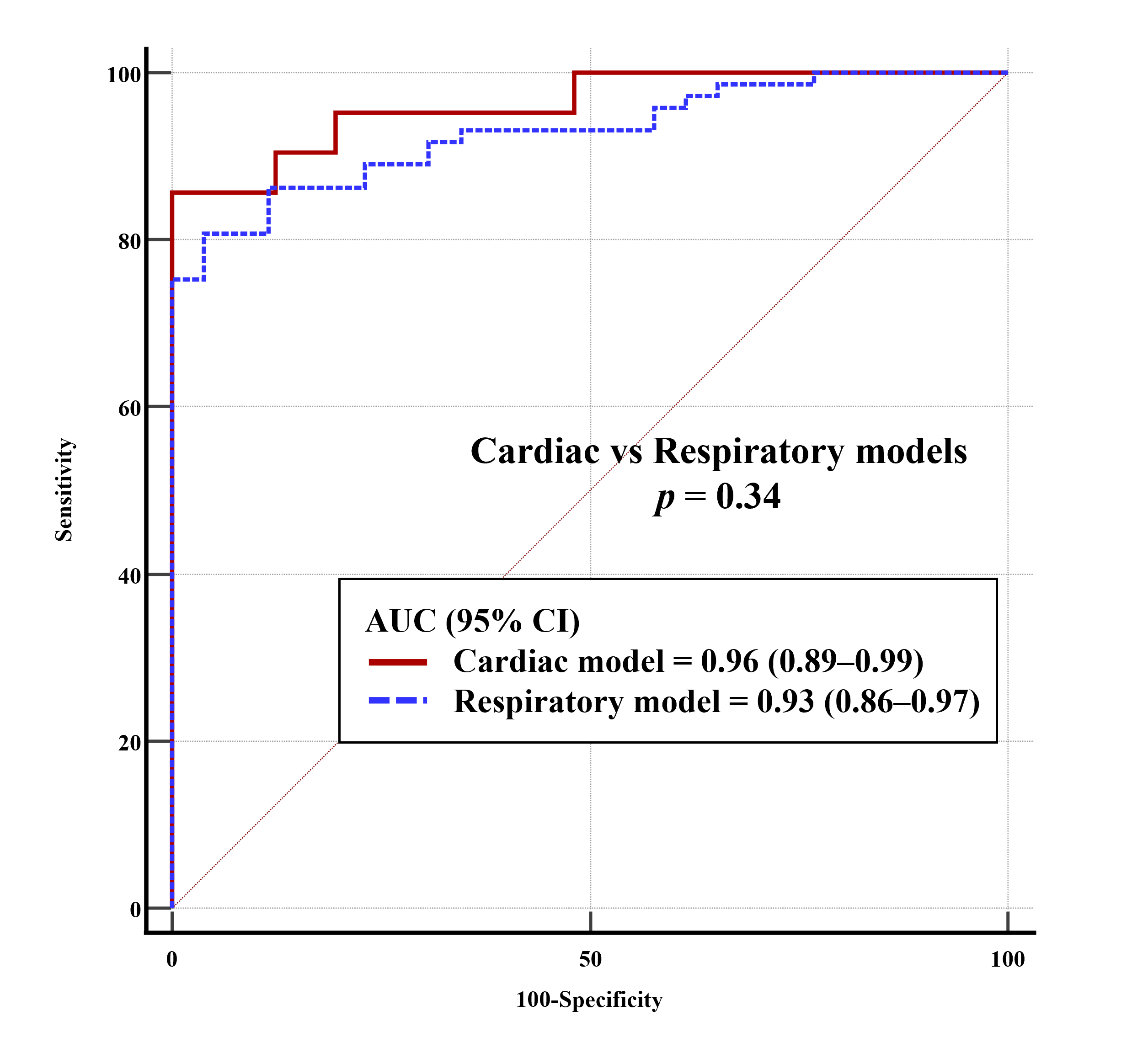
Figure S7. Receiver operating characteristic (ROC) curves for the final multivariable prediction model for poor neurological outcome, stratified by arrest etiology.**

The model showed excellent discrimination for predicting poor neurological outcome in cardiac and respiratory etiologies, with AUC values of 0.96 (95% CI: 0.89–0.99) and 0.93 (95% CI: 0.86–0.97). No significant difference (DeLong *P* = 0.34). These ROC curves demonstrate robust performance (Additional file 1, Table S4). Both subgroups demonstrated good calibration (Hosmer–Lemeshow *P*>0.2) and low Brier scores, indicating robust overall model performance.

Abbreviations: AUC, area under the receiver operating characteristic curve; CI, confidence interval; DW-MRI, diffusion-weighted magnetic resonance imaging


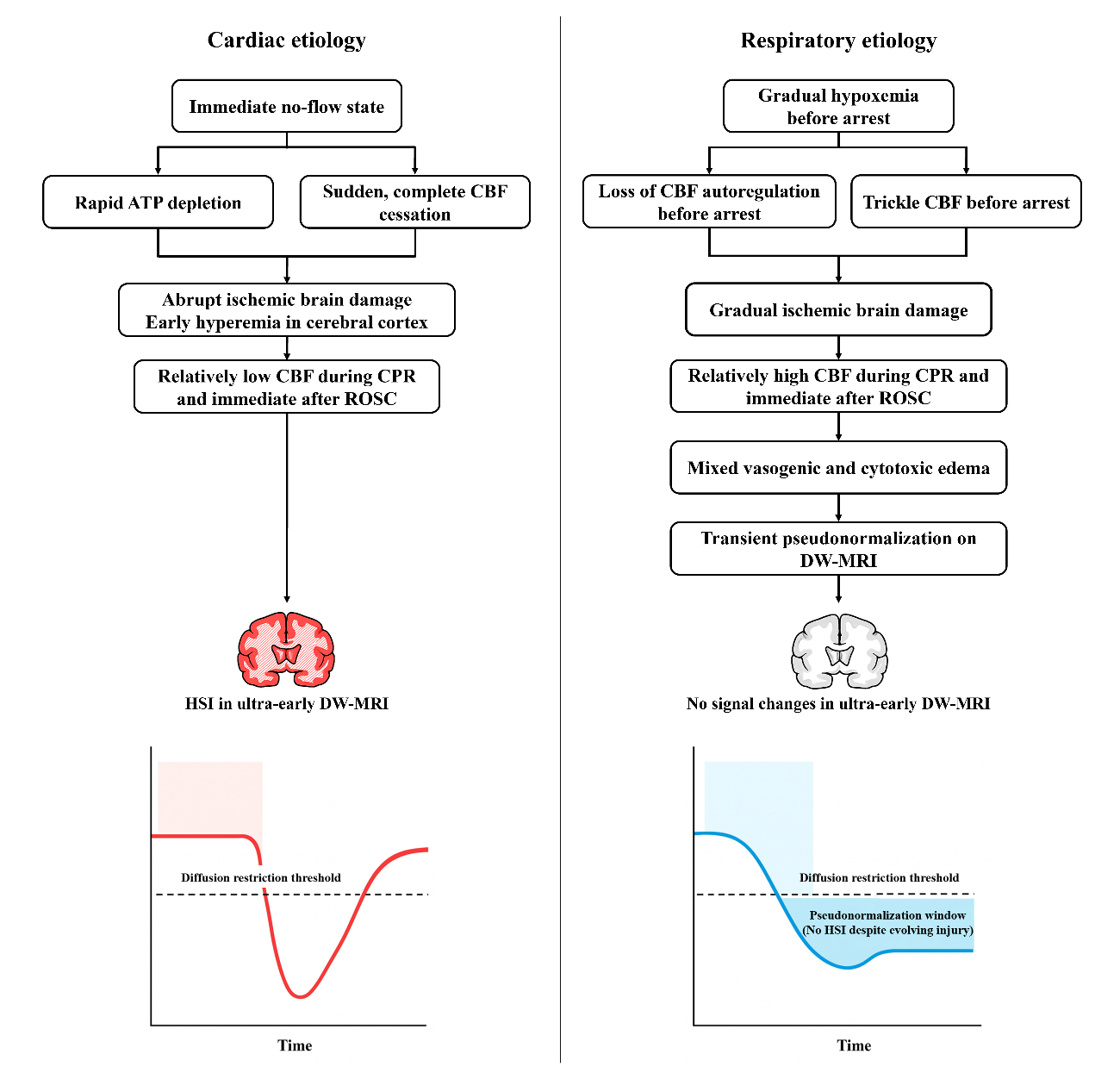
**Figure S8. Conceptual schematic illustrating pathophysiological, perfusion, and DW-MRI differences between cardiac and respiratory arrest etiologies**

The left panel (cardiac etiology) depicts the consequences of an abrupt no-flow state with rapid ATP depletion and sudden CBF cessation, leading to early ischemic injury and modest early hyperemia confined to the cerebral cortex. Owing to severe myocardial dysfunction and a prolonged true no-flow interval, CBF remains relatively low during CPR and immediately after ROSC, resulting in early, relatively homogeneous cytotoxic edema that becomes detectable as HSI on ultra-early DW‑MRI. The corresponding ADC trajectory demonstrates a rapid decline that crosses the diffusion‑restriction threshold shortly after ROSC, leaving little opportunity for any transient pseudonormalization.

The right panel (respiratory etiology) illustrates the progressive development of hypoxemia, loss of cerebrovascular autoregulation, and persistence of “trickle‑flow” perfusion before complete arrest. This combination produces relatively higher CBF during CPR and early ROSC and generates mixed vasogenic and cytotoxic edema with delayed, regionally heterogeneous ischemic injury. During this phase, residual perfusion and vasogenic components can partially normalize the net diffusion signal, creating a transient pseudonormalization window where DW‑MRI appears near‑normal despite evolving tissue injury. Consequently, ultra-early DW‑MRI frequently shows fewer or absent visible diffusion‑restriction findings in respiratory arrest, with diffusion restriction becoming apparent only later as the ADC trajectory gradually declines and crosses the diffusion‑restriction threshold.

Overall, the schematic highlights that cardiac etiology produces early, synchronous diffusion restriction with minimal pseudonormalization, whereas respiratory etiology results in delayed, heterogeneous restriction with a period of transient pseudonormalization, consistent with distinct etiology-specific perfusion–metabolic mechanisms.

Abbreviations: ATP, adenosine triphosphate; CBF, cerebral blood flow; CPR, cardiopulmonary resuscitation; ROSC, return of spontaneous circulation; HSI, high signal intensity; DW-MRI, diffusion-weighted magnetic resonance imaging; ADC, apparent diffusion coefficient
